# Heterogeneity of SARS-CoV-2 immune responses after the nationwide Omicron wave in China

**DOI:** 10.1128/spectrum.01117-24

**Published:** 2024-09-17

**Authors:** Jing Wu, Mingzheng Jiang, Jiwei Li, Xiaoyi Hu, Qiuyue Long, Shixu Song, Hongli Ye, Yukun He, Xinqian Ma, Wenyi Yu, Xi Chen, Lili Zhao, Fangfang Wu, Xiaoyong Chen, Jianshi Zheng, Minghui Wang, Binghan Zheng, Shuoqi Yang, Liang Bu, Qin Chen, Ke Li, Yali Zheng, Zhancheng Gao

**Affiliations:** 1 Department of Respiratory, Critical Care and Sleep Medicine, School of Medicine, Xiamen University, Xiang'an Hospital of Xiamen University, Xiamen, Fujian, China; 2 Institute of Chest and Lung Diseases, Xiang'an Hospital of Xiamen University, Xiamen, Fujian, China; 3 Department of Respiratory and Critical Care Medicine, Peking University People’s Hospital, Beijing, China; 4 Department of Thoracic Surgery, School of Medicine, Xiamen University, Xiang'an Hospital of Xiamen University, Xiamen, Fujian, China; 5 Department of Cardiovascular Medicine, School of Medicine, Xiamen University, Xiang'an Hospital of Xiamen University, Xiamen, Fujian, China; 6 Department of Critical Care Medicine, School of Medicine, Xiamen University, Xiang’an Hospital of Xiamen University, Xiamen, Fujian, China; University of Georgia, Athens, Georgia, USA

**Keywords:** COVID-19, humoral immunity, Omicron, inactivated vaccine, repeated vaccination

## Abstract

It remains unclear how previous infections and vaccinations influenced and shaped heterogeneous immune responses against Omicron and its variants in diverse populations in China. After the national wave of Omicron in early 2023, we evaluated serum levels of neutralizing antibodies (nAbs) against Omicron (B.1.1.529) and its variants (BA.5, BF.7, and CH1.1) in 33 COVID-19 convalescents and 40 uninfected vaccinees, using vesicular stomatitis virus-based pseudovirus neutralizing assay. In addition, we followed 34 Delta convalescent patients to compare their immune responses against Omicron before (late 2021) and after the Omicron wave (early 2023). NAbs at the acute phase of the disease were investigated in 50 Omicron inpatients, including 24 vaccinated and 26 unvaccinated patients. Among them, nasal mucosal IgA levels were measured in 42 subjects. Compared to vaccination, breakthrough infections significantly increased the breadth and magnitude of serum nAbs and mucosal IgA levels against Omicron variants. Exposure to Omicron but not Delta elicited stronger pan-Omicron responses. In Omicron inpatients, nAbs continued to rise as vaccination doses increased. However, in both vaccinees and convalescents, a fourth dose vaccination did not elicit higher nAbs against Omicron. Furthermore, nAbs against Omicron variants lasted longer than nAbs against WT SARS-CoV-2. Breakthrough infections of Omicron variants elicited specific immune responses against Omicron compared to vaccination and Delta infection. Although repeated vaccination revealed limited impacts on serum nAbs, populations at high risk of hospitalization may still benefit from continued vaccination.

**IMPORTANCE:**

The study described the specific humoral immunity against Omicron and its variants (BA.5, BF.7, and CH1.1) in diverse populations, including Delta-positive convalescent patients, Omicron-infected patients with a previous or current confirmed Delta infection, Omicron-positive patients, and healthy controls. In addition, we followed Delta convalescents for 1 year to evaluate the effect of a booster vaccine, breakthrough infection, and reinfection. Nasal mucosal IgA levels against SARS-CoV-2 were also examined. The findings of this study demonstrated the varied responses of individuals in different states following the outbreak of Omicron, highlighting the potential advantages of ongoing immunization for groups that are more vulnerable and have a greater likelihood of being hospitalized.

## INTRODUCTION

While the World Health Organization (WHO) has announced that the COVID-19 epidemic no longer comprises a “Public Health Emergency of International Concern” (1), SARS-COV-2 will continue to spread at different levels in the communities with outbreaks here and there. The pathogenicity of the Omicron variant was lower compared to that of other variants of concern (VOCs) (2). However, Omicron’s increased transmissibility is attributed to its evasion of vaccine-elicited neutralizing antibodies, superior invasion of nasal epithelia, and resistance to cell-intrinsic barriers (3). Therefore, for susceptible populations such as the elderly, individuals with chronic conditions, and those with weakened immune systems, the ongoing transmission of the virus still presents a significant health risk. New variants of concern will continue to emerge, which could be more transmissible and pathogenic resulting in more severe manifestations. Global priorities continue to focus on limiting the spread of SARS-CoV-2 and preventing severe cases of COVID-19.

China’s complete vaccination rate has reached 90.26%, with over 800 million individuals receiving initial booster shots as of 11 November 2022 (4). In addition, Omicron subvariants BA.1, BA.2, BA.4/5, and BF.7 have been actively replacing other VOCs and local variants to become the most dominant variant in many parts of the world (5). Besides, Omicron variants have caused national waves since China’s dynamic zero-COVID-19 policy was canceled on 7 December 2022. The impact of prior infections and immunization on humoral immune responses remains unclear. Furthermore, intranasal mucosal vaccines including dNS1-RBD have demonstrated their distinct advantages in stimulating hormonal, cellular, and mucosal responses while also preventing severe infection (6). The heterogeneity of immune responses against SARS-CoV-2 has become considerably more complex over time in China and has posed a significant public health obstacle in vaccine strategies.

In addition, concerns regarding the safety and effectiveness of repeated vaccinations have emerged. Gao et al. found that after BA.5/BF.7 breakthrough infection, neutralizing antibodies (nAbs) against the Omicron BA.2, BA.4, and BA.5 variants were significantly lower in people who received a three-dose regimen inactivated vaccine, compared to those who received a two-dose regimen vaccine (7). A comparable phenomenon was not observed in other reports (8), a third dose of inactivated vaccine could still significantly recall and enhance antibody responses against Omicron variants. The controversial results prompted us to clarify the effects of booster doses of inactivated vaccines on humoral immunity.

In this study, we examined the specific humoral and nasal mucosal immune responses against Omicron and its variations (BA.5, BF.7, and CH1.1) in varied groups of subjects. We discovered that natural infections elicited higher neutralizing activities against Omicron and its variants, compared to vaccinations. COVID-19-hospitalized patients who received a fourth booster vaccine showed higher nAbs against Omicron variants in both strength and breadth, compared to those who received 2–3 doses. Furthermore, all subjects exhibited measurable levels of nasal mucosal IgA against Omicron and its variations, although these levels did not correlate with humoral immune activity.

## MATERIALS AND METHODS

### Human subjects and sample collection

Serum samples from 33 COVID-19 convalescents, including Delta infected (*n* = 7), Delta and subsequent BA.5/BF.7 reinfected (*n* = 7), and BA.5/BF.7 infected (*n* = 19), as well as healthy controls (*n* = 40), were examined to comprehend the variety of immune responses within the community. In all, 50 Omicron inpatients were also enrolled to elucidate the immune status at the acute phase. In addition, a 1-year follow-up study including 34 individuals was conducted to further assess the impact of a booster vaccine, breakthrough infection, and reinfection. A total of 42 nasal swab samples were obtained, including 9 healthy vaccinees, 16 BA.5/BF.7 convalescents, and 17 BA.5/BF.7 inpatients. The infection status was confirmed *via* polymerase chain reaction tests. The vaccination records and other demographic data were required. Serum samples were isolated and stored at −80°C until analysis. For COVID-19 convalescents, serum samples were collected within 2 months after infection. For inpatients, serum samples were obtained within 72 h of admission (Table 1). Samples were heat-inactivated at 56°C for 30 minutes before further analysis in a BSL2 laboratory.

**TABLE 1:** Characteristics of all individuals*^c^*

	Total (2023) (*n* = 103)	Delta convalescent (2021.12) (*n* = 36)	Delta convalescent Followed for 1 year (*n* = 14)		Healthy donors (2021.12) (*n* = 48)	Healthy donors Followed for 1 year (*n* = 20)		Other healthy donors (2023) (*n* = 10)	Omicron infected (*n* = 59)	
									Omicron convalescent (2023) (*n* = 9)	Omicron inpatients (2023) (*n* = 50)
			Uninfected (2023) (*n* = 7)	Omicron infected (2023) (*n* = 7)		Uninfected (2023) (*n* = 10)	Omicron infected (2023) (*n* = 10)			
Gender										
Female, n (%)	43 (41.7%)	17 (47.2%)	5 (71.4%)	5 (71.4%)	25(52.1%)	3 (30%)	4 (40%)	7 (70%)	4 (40%)	12 (24%)
Vaccination status										
Unvaccinated, n (%)	26 (25.2%)	21 (58.3%)	-	-	-	-	-	-	-	26 (52%)
Age <18, n (%)	_^-^_^c^	8 (22.2%)	-	-	-	-		-	-	-
18–65, n (%)	7 (6.8%)	10 (27.8%)	-	-	-	-		-	-	7 (14%)
>65, n (%)	19 (18.4%)	3 (8.3%)	-	-	-	-		-	-	19 (38%)
One dose, n (%)	4 (3.9%)	-	-	1 (14.3%)	-	-	-	-	-	3 (6%)
Age <18, n (%)	-	-	-	-	-	-	-	-	-	-
18–65, n (%)	1 (1%)	-	-	1 (14.3%)	-	-	-	-	-	-
>65, n (%)	3 (2.9%)	-	-	-	-	-	-	-	-	3 (6%)
Two dose, n (%)	15 (14.6%)	15 (41.7%)	1 (14.3%)	2 (28.6%)	23(47.9%)	-	1 (10%)	1 (10%)	-	10 (20%)
Age <18, n (%)	-	-		-	-	-	-	-	-	-
18–65, n (%)	7 (6.8%)	12 (33.3%)	1 (14.3%)	2 (28.6%)	23(47.9%)	-	1 (10%)	1 (10%)	-	2 (4%)
>65, n (%)	8 (7.8)	3 (8.3%)	-	-	-	-	-	-	-	8 (16%)
Three dose, n (%)	32 (31.1%)	-	6 (85.7%)	4 (57.1%)	25 (52.1%)	4 (40%)	3 (30%)	3 (30%)	4 (44.4%)	8 (16%)
Age <18, n (%)	-	-	-	-	-	-	-	-	-	-
18–65, n (%)	25 (24.3%)	-	6 (85.7%)	4 (57.1%)	25 (52.1%)	4 (40%)	3 (30%)	3 (30%)	4 (44.4%)	1 (2%)
>65, n (%)	7 (6.8%)	-	-	-	-	-	-	-	-	7 (14%)
Four dose/hybrid vaccination^*a*^, n (%)	26 (25.2%)	-	-	-	-	6 (60%)	6 (60%)	6 (60%)	5 (55.6%)	3 (6%)
Age <18, n (%)	-	-	-	-	-	-	-	-	-	-
18–65, n (%)	23 (22.3%)	-	-	-	-	6 (60%)	6 (60%)	6 (60%)	5 (55.6%)	-
>65, n (%)	3 (2.9%)	-	-	-	-	-	-	-	-	3 (6%)
Nasal swab, n (%)	42 (40.8%)	-	-	-	-	4 (40%)	8 (80%)	5 (50%)	8 (88.9%)	17 (34%)
Time interval^*b*^ (days)	-	31.5 (25–35)	279 (279–305)	96 (92.5–106.5)	102.5 (54.8–190.8)	307.5 (47–421.3)	47.5 (41.3–49.3)	61.5 (49.3–138.5)	49 (49–50)	1 (1-1)

^*a*^ Three doses of inactivated vaccine with additional nasal spray vaccine.

^*b*^ Time interval for healthy donors indicates the period between their last vaccination and blood sample collection. For Delta convalescents and Omicron convalescents, it indicates the period between infection and blood sample collection. For Omicron inpatients, it indicates the period between hospitalization and blood sample collection. Data are presented as n (%) or median (IQR).

^*^c^*^ "-” indicates not applicable to this column.

### Cultured cell lines

We used HEK293T cells (Procell) to establish an ACE2 & TMPRSS2 stable expressing cell line (293T-ACE2-TMPRSS2). The cells were cultivated in Dulbecco’s modified Eagle medium (DMEM) supplemented with 10% fetal bovine serum (FBS) and incubated at 37°C in a 5% CO_2_ setting. As we previously reported (9), plasmids encoding angiotensin-converting enzyme 2 (pLV-ACE2-3xFLAG-IRES-puro, HedgehogBio Science and Technology Ltd.) and transmembrane serine protease 2 (pLV-TMPRSS2-GFP, Sino Biological) were co-transfected into 293T cells and the expression of ACE2 and TMPRSS2 were confirmed *via* western blot.

### SARS-CoV-2 pseudovirus neutralization assay

The pseudoviruses encoding the S protein of various SARS-CoV-2 variants, including the wild type (WT), Omicron (B.1.1.529), and its subvariants (BA.5, BF.7, and CH1.1), were acquired from Vazyme Biotech. The luciferase gene was integrated into the VSV vector and can be expressed after infection with the pseudotyped virus. The TCID50 (50% tissue culture infectious dose) value (10) was used to quantify virus concentration according to the manufacturer’s instructions. Neutralization assays were performed on 293T-ACE2-TMPRSS2 cells. Serum samples were 1:16 diluted, followed by a threefold serial dilution. The diluted sera (50 µL) were mixed with pseudotyped SARS-CoV-2 viruses (650 TCID50 per well) in 96-well plates and incubated at 37°C and 5% CO_2_ for 1 h. Afterward, the mixture was co-cultured with the 293T-ACE2-TMPRSS2 cells (20,000 per well) for the next 24 h (11).

The chemiluminescence signals were quantified in relative luminescence units (RLU) using a Bright GloTM luciferase assay system with a GloMax Navigator Microplate Luminometer. The neutralizing titers (NAT50) were defined as the 50% inhibitory dilution (ID50), which was calculated with the highest dilution of plasma that resulted in a 50% reduction of relative light units compared with virus control. NAT50 below 16 was considered negative. The NAT50 values within groups were summarized as geometric mean neutralizing titers (GMT) with a 95% confidence interval (95% CI).

### SARS-Cov-2-specific IgA enzyme-linked immunosorbent assay

Each nasal swab was immersed in 1 mL of phosphate-buffered saline (PBS, Thermo Fisher). Enzyme-linked immunosorbent assay (ELISA) plates were coated overnight at 4°C with SARS-CoV-2 receptor binding domain (RBD) protein (1 g/mL in PBS, Vazyme Biotech). After standard cleansing and sealing for 2 h at 37°C, diluted nasal swab samples (1:2-1:128, 2-fold serial diluted) were added to wells and incubated for 1 h at the same temperature. Each well was treated with 100 μL of HRP-labeled anti-human IgA monoclonal antibody (Thermo Fisher) and incubated at 37°C for 1 h. A tetramethylbenzidine (TMB) chromogenic agent solution (Thermo Fisher) was used as the substrate and the optical density (OD) values were measured at 450 nm absorbance. Subject operating characteristic (ROC) analysis on the OD values of various individuals was performed and the area under the curve (AUC) to represent the nasal mucosal IgA antibody concentration for each individual was calculated.

### Statistical analysis

Statistical analyses were performed using GraphPad Prism 9.5.1 and SPSS 29.0. The Pearson χ^2^ test or Fisher’s exact test was used for comparisons between the two groups. One-way ANOVA with Tukey’s multiple comparison test was used to compare differences among multiple groups. Statistical significance was defined as *P* < 0.05. The error bars in all figures represent either a 95% confidence interval or one standard deviation where indicated.

## RESULTS

### Study design and participants’ characteristics

**Fig 1:**
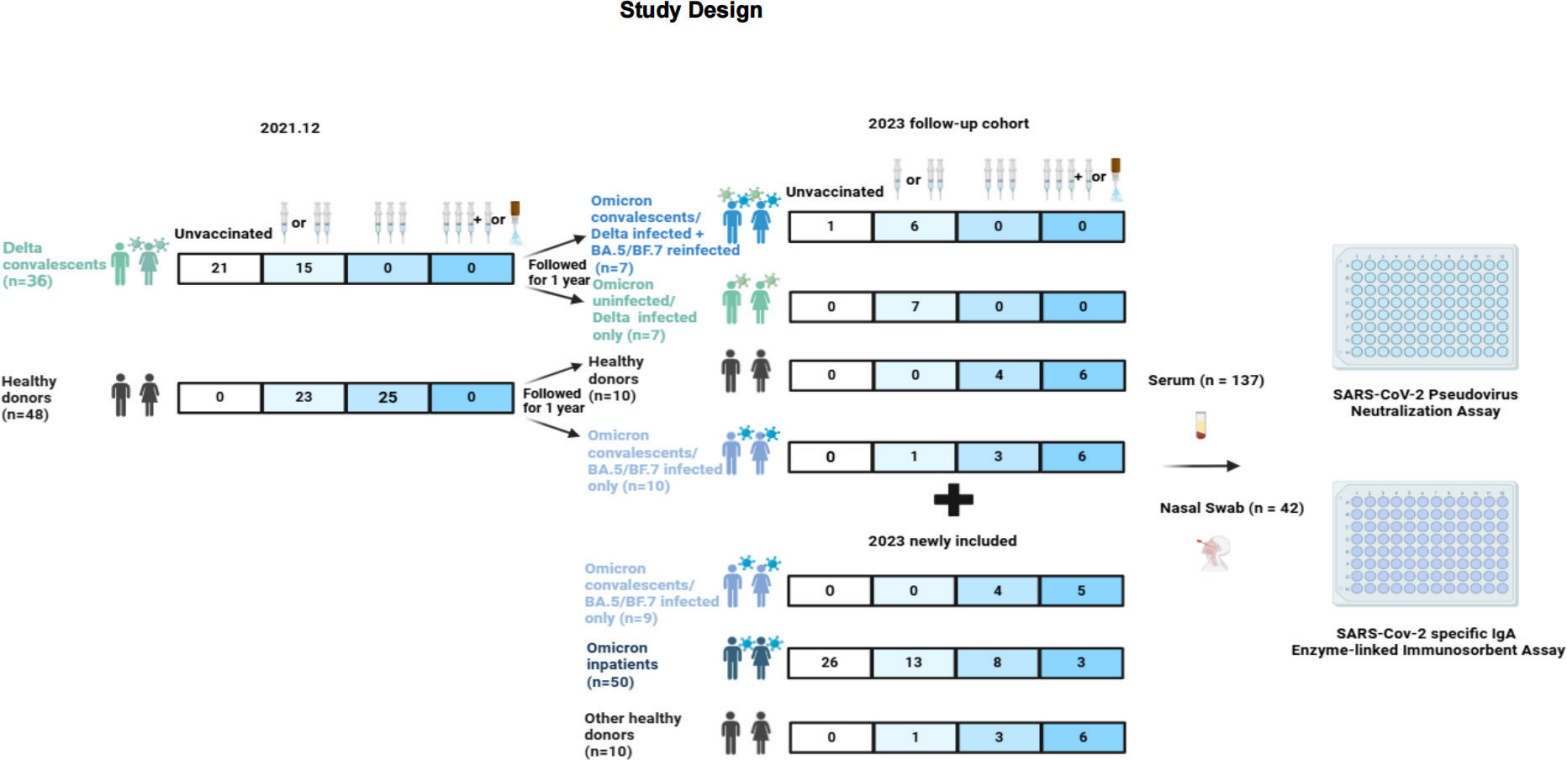
The various cohorts of populations. The number of unvaccinated, one-, two-dose, three-dose, and four-dose or hybrid vaccinated (three-dose inactivated vaccine with nasal spray vaccine) participants in different groups. Collect serum to evaluate the level of neutralizing abilities (nAbs) *via* pseudovirus neutralization assay, and collect nasal swabs to evaluate the immune level of nasal mucosa *via* Elisa assay.

A total of 103 participants were enrolled. Nine Omicron convalescents, 50 Omicron inpatients, and 10 healthy controls were included from January 2023 to March 2023. In all, 34 cases were enrolled during 2021 when a local Delta outbreak happened in China. The participants were followed up for 1 year and 14 out of them contracted Omicron subvariants in early 2023. The majority of Omicron inpatients were aged over 65 (42/50, 84%). Detailed information can be found in Fig. 1 and Table 1.

Among the participants, 27 (26.2%) remained unvaccinated, while 50 (48.5%) received 2–3 doses of inactivated virus vaccines (BBIBP-CorV, Sinopharm, Beijing CNBG, or CoronaVac, SinoVac). In addition, 26 participants (25.2%) received nasal vaccination (dNS1-RBD, Beijing Wantai BioPharm) after a booster dose. In all, 42 individuals, consisting of 7 unvaccinated, 15 vaccinated, and 11 nasal vaccinated COVID-19 participants and 9 vaccinated healthy donors, underwent nasal swab testing to measure mucosal IgA levels against WT, Omicron, and its variants.

### Diminished neutralization against Omicron and its subvariants

**Fig 2:**
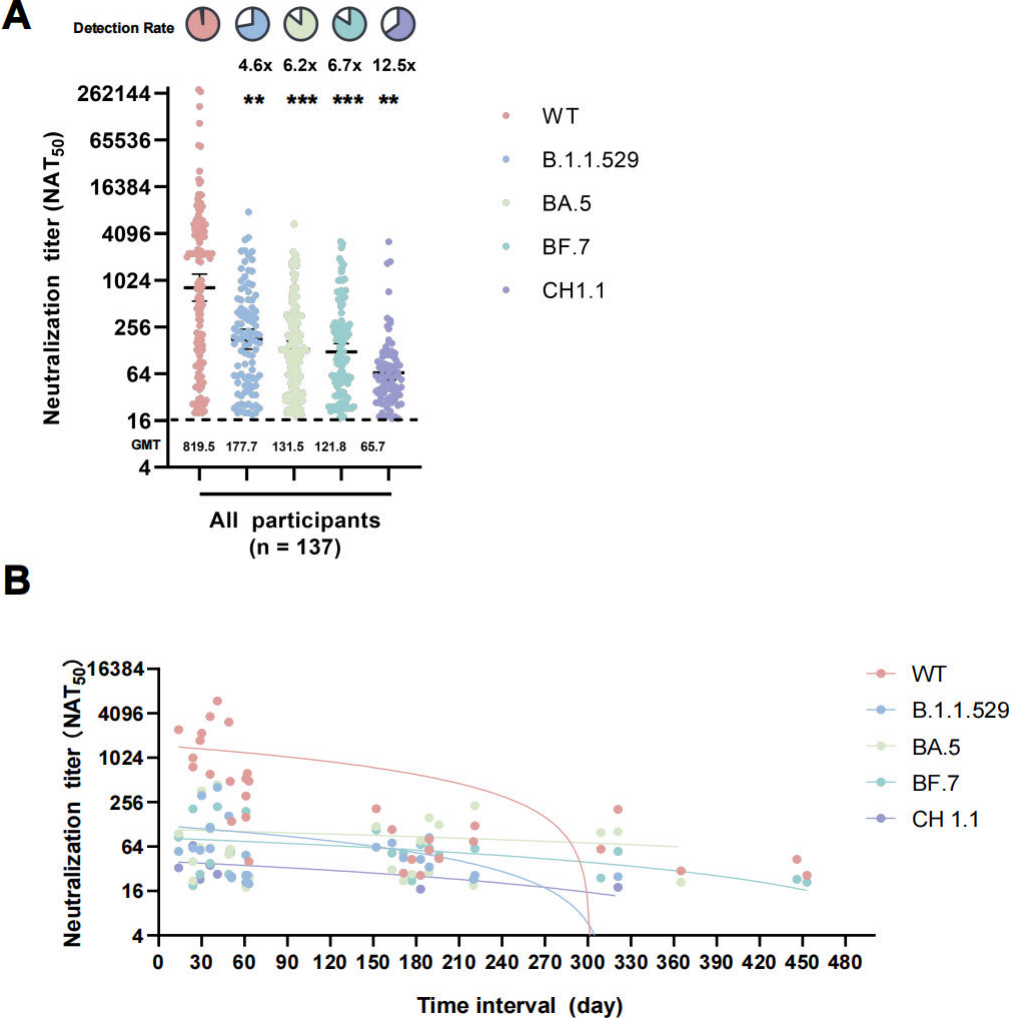
Reduced neutralization against Omicron and its Subvariants. (**A**) The 50% neutralization titers (NAT50) were determined *via* VSV pseudovirus neutralization assay against WT (pink dots), B.1.1.529 (blue dots), BA.5 (green dots), BF.7 (dark green dots), and CH1.1 (purple dots) variants in all samples. (**B**) NAbs showed a sharp decline between 180 and 300 days across all Omicron subvariants and WT in healthy donors. Time interval indicated the period between the last vaccination and blood sample collection in healthy donors. Different colored dots and lines represent the nAbs and correlation curves of WT (pink), B.1.1.529 (blue), BA.5 (green), BF.7 (dark green), and CH1.1 (purple). Data are presented as scatter dot plots with error bars indicating the geometric mean titers (GMT) with a 95% CI. The GMT values are shown on the axis X. Fold changes and *P* values of GMTs compared to WT by Omicron variants are shown at the top of each group. *P < 0.05, **P < 0.01, ***P < 0.001, ****P < 0.0001. Pie charts show the proportion of individuals within each group that had detectable neutralization against the indicated SARS-CoV-2 variants. All neutralization assays were conducted in biological duplicates.

We analyzed a total of 137 serum samples collected from the above 103 participants. We first determined the general immune responses against WT, and Omicron (B.1.1.529) and its variants (BA.5, BF.7, and CH1.1). The findings demonstrated a substantial decline in both the breadth and potency of nAbs (Fig. 2A; Table S1). Neutralization was detected in 97.1% (133/137) of cases against the WT strain. For the B.1.1.529, BA.5, BF.7, and CH1.1 strains, neutralizations were discovered in 72.3% (99/137), 85.4% (117/137), 83.9% (115/137), and 63.5% (87/137) of cases, respectively. The neutralization titers (GMT) against the B.1.1.529 variant reduced by a factor of 4.6 (relative to the WT) in the pseudovirus experiment (*P* < 0.01), with a GMT of 819.5 (95% CI 549.3–1222.4) for the WT. Similarly, decreases of 6.2-fold (*P* < 0.001), 6.7-fold (*P* < 0.001), and 12.5-fold (*P* < 0.01) were seen in GMT against BA.5, BF.7, and CH1.1, respectively. The results indicate a general reduction in neutralizing activities of sera against Omicron variants. The fall in effectiveness followed the order: CH1.1 >BF.7 >BA.5 >B.1.1.529. Notably, there was a significant decline in neutralizing antibodies (nAbs) against the WT at around 180 days during the period between the last vaccination and blood sample collection in healthy donors. NAbs against Omicron B.1.1.529 showed a decrease at approximately 210 days, whereas the nAbs against Omicron variants BA.5, BF.7, and CH1.1 remained present for an extended period and did not drop until roughly 300 days (Fig. 2B). Thus, the duration of nAbs against Omicron variant seems to be longer compared to nAbs against WT.

### Breakthrough infection significantly enhanced neutralizing activities against Omicron variants

**Fig 3:**
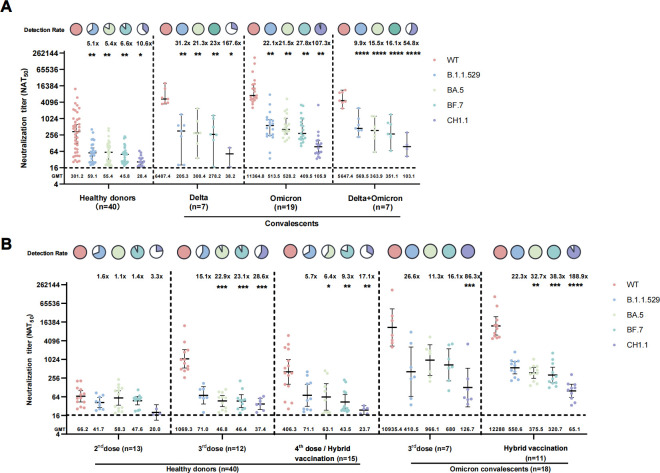
NAbs levels in different vaccination and infection backgrounds. (**A**) NAbs against WT and Omicron variants in healthy donors, Delta convalescents, Omicron convalescents, and Delta and subsequent Omicron variant reinfected convalescents. (**B**) NAbs against WT and Omicron variants in serum from different vaccination backgrounds of Omicron convalescents and healthy donors. Data are presented as scatter dot plots with error bars indicating the GMT with a 95% CI. The GMT values are shown on the axis X. Fold changes and *P* values of GMTs compared to WT by Omicron variants are shown at the top of each group. *P < 0.05, **P < 0.01, ***P < 0.001, ****P < 0.0001. Pie charts show the proportion of individuals within each group that had detectable neutralization against the indicated SARS-CoV-2 variants. All neutralization assays were conducted in biological duplicates.

We divided COVID-19 convalescents into three groups, BA.5/BF.7 infected only, Delta infected only, and Delta infected and subsequent BA.5/BF.7 reinfected. Then we compared the nAbs among the groups (Fig. 3A; Table S2). Breakthrough infections significantly increased neutralizing activities against WT and Omicron variants. Although detection rates of WT were 100% in all four groups, the GMTs against WT were 18.7–37.7 times higher in COVID-19 convalescents than in vaccinees (*P* < 0.01). The nAbs against Omicron variants were present in 28.6%–100% of the COVID-19 convalescents and 35%–100% of vaccinees. However, the GMTs against Omicron variants of COVID-19 convalescents were 1.3–9.6 times higher than that of vaccinees.

Given that CH1.1 was only detected in imported cases in China mainland (12), we chose CH1.1 as a representative example of a newly emerging variant. Among individuals who have recovered from COVID-19, the nAb against CH1.1 increased by 1.3–3.7 times compared to those who received the vaccines. Moreover, infection with BA.5/BF.7 elicited stronger immune responses against Omicron variants compared to Delta infection. The GMT against Omicron variants of BA.5/BF.7 convalescents enhanced by a factor of 1.5–2.8 compared to Delta convalescents. The detection rate of CH1.1 even reached 95% in Omicron convalescents while it was only 28.6% in Delta convalescents. Although the GMTs of prior-naïve Omicron convalescent sera against WT and BA.5 was 1.5-fold to two-fold higher than that of prior-Delta-infected Omicron convalescent, the GMTs against B.1.1.529, BF.7and CH1.1 were similar between the two subgroups, as shown in Fig. 3A.

### A fourth dose of an inactivated vaccine or an extra nasal vaccine showed limited effects on elevating serum nAbs

Gao et al. reported that three doses of inactivated vaccine dampen nAbs against Omicron variants in BA.5/BF.7 breakthrough infection compared to two doses (7). Thus, we compared the nAbs in cases with various vaccination backgrounds. As shown in Fig. 3B and Table S3, in healthy vaccinees, although the 3–4 doses resulted in a 16.2-fold increase in GMTs in neutralizing activity in sera against WT, compared to the fully vaccinated healthy donors (*P* < 0.0001), little increase in the breadth and levels of nAbs against Omciron and its variants were observed. For CH1.1, the GMTs and detection rates of vaccinees receiving two doses, three doses, and four doses/additional nasal vaccine were 20 (23.1%), 37.4 (58.3%), and 23.7 (33.3%), respectively. In convalescents, the detection rates of WT and Omicron variants were equivalent in the three-dose subgroup (100%) and hybrid vaccination subgroup (90.9%–100%), and the GMTs against Omicron variants in the two subgroups ranged from 126.7 to 966.1 and 65.1 to 550.6. Neither subgroup observed an obvious increase or downward trend in nAbs against Omicron variants as the number of vaccines increased.

In addition, we evaluated the nAb levels against Omicron B.1.1.529 in healthy vaccinees who received a booster dose compared to fully vaccinated subjects using our previous data (8). As shown in Fig. S1B and Table S4, a booster dose resulted in a 1.4-fold and 8.2-fold increase in the neutralizing abilities against B.1.1.529 and WT (*P* < 0.05), respectively. Above all, there was no significant decrease in the levels of nAbs as the number of vaccinations rose.

### Longitudinal evaluation of the cross-neutralizing efficacy

In addition, we contrasted 34 individuals’ nAbs before and after a year of follow-up, including 20 healthy vaccinees and 14 delta convalescents in 2021. In all, 20 healthy donors received an additional vaccine during the follow-up. Among them, 10 remained uninfected in 2023 and their nAbs levels against WT increased from 193.2 to 649.1, and the levels against Omicron variants increased from 28.2 to 67 to 27–112. The other 10 suffered BA.5/BF.7 infection in early 2023, the GMT against WT peaked at 10915.3 (95% CI 4728–25198), which was 40.2-fold higher than that of the uninfected period (GMT 271.8, 95% CI 76.9–961.2). Likewise, GMTs against Omicron and its subvariants were also increased by 2.8-fold to 10.1-fold compared to uninfected intervals. The detection rates of WT, B.1.1.529, BA.5, and BF.7 all increased to 100% after infection, notably, while the detection rate of CH1.1 increased from 50% to 90%.

**Fig 4:**
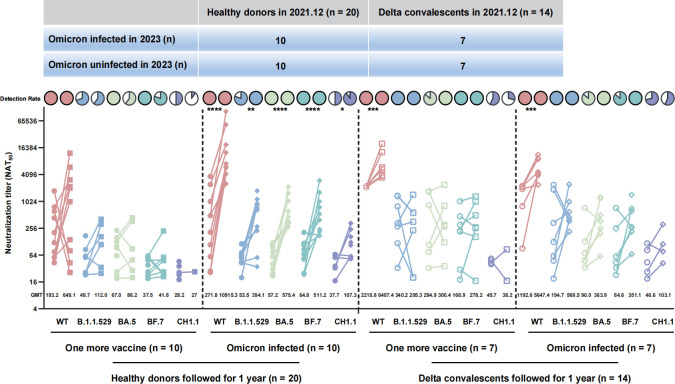
NAbs increased in the same followed-up individuals after receiving more vaccines or being infected with Omicron. At the end of 2021, 34 individuals were followed up for 1 year. In 20 healthy donors (solid circles), 10 participants were infected with BA.5/BF.7 (solid diamond) and 10 participants received one more vaccine (solid square). In 14 Delta convalescents (hollow circle), seven participants were infected with BA.5/BF.7 (hollow diamond) and seven participants received one more vaccine (hollow square). The GMT values are shown at the bottom of the dots. Pie charts show the proportion of each group that had detectable IgA levels against the indicated SARS-CoV-2 variants. The *P* values of GMTs at different times are shown at the top of each group. *P < 0.05, **P < 0.01, ***P < 0.001, ****P < 0.0001. The trend of nAb changes in the same person before and after a 1-year follow-up are represented by lines of different colors: WT (pink), B.1.1.529 (blue), BA.5 (green), BF.7 (dark green), and CH1.1 (purple).

In 14 Delta convalescents, seven suffered BA.5/BF.7 infection and seven received an additional dose of vaccine. The GMTs against WT both increased after Omicron infection and vaccination, with 2.9-fold (*P* < 0.001) and 4.7-fold (*P* < 0.001) elevation, respectively. The GMTs against Omicron subvariants, however, appeared to have minimal changes in vaccinees but increased by 2.2–4.2 folds after Omicron infection (Fig. 4). The detection rates were all increased to 100% after infection or receiving more vaccines except for CH1.1. The results indicated that both infection and increasing vaccination can lead to enhanced levels of neutralization abilities.

### Assessment of the neutralizing abilities in severely ill individuals

**Fig 5:**
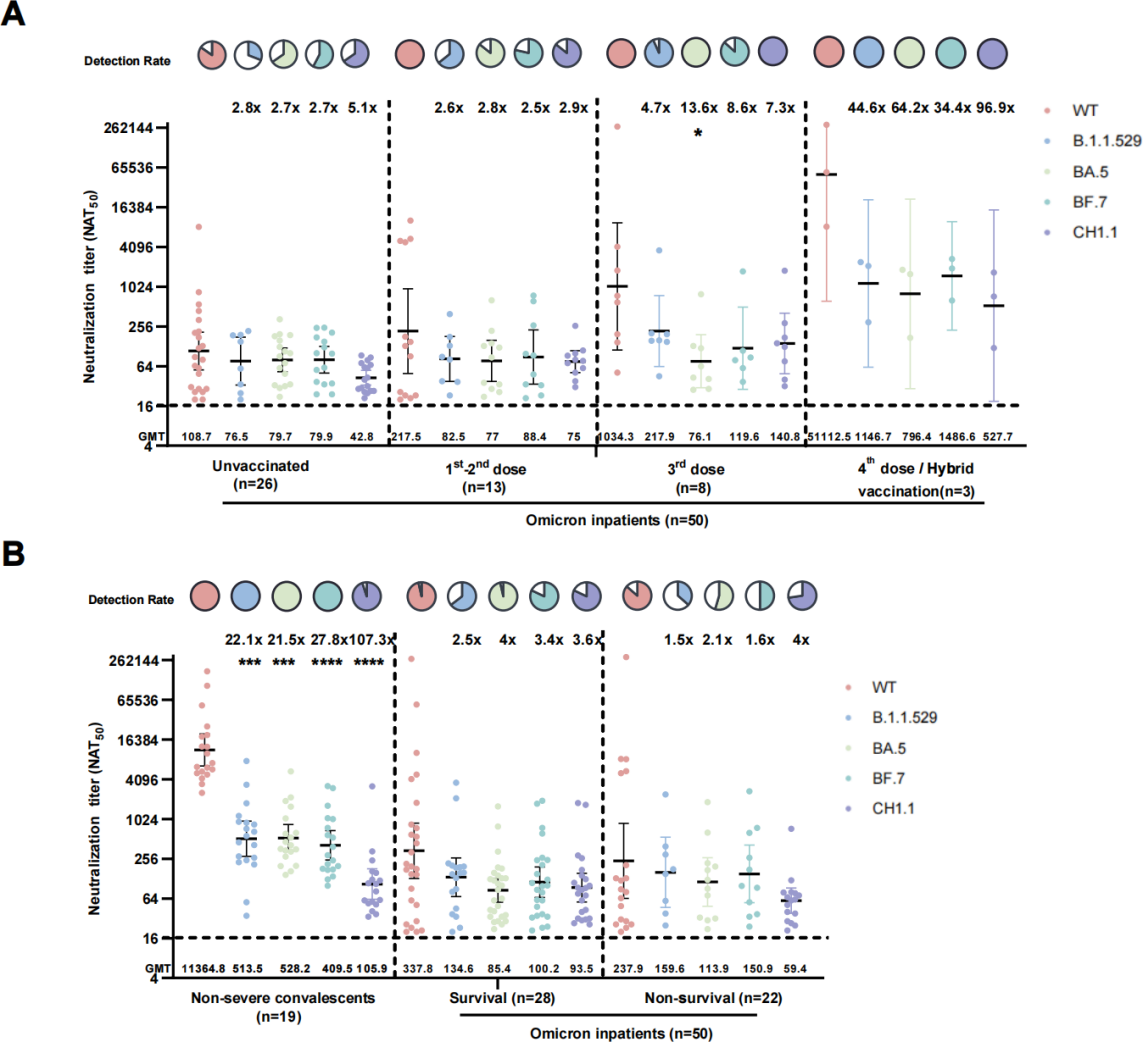
NAbs against WT and Omicron variants in different disease severity subgroups. (**A**) NAbs against WT and Omicron variants in serum from different vaccination backgrounds of inpatients. (**B**) NAbs against WT and Omicron variants in Omicron convalescents, survival inpatients, and non-survival inpatients. Data are presented as scatter dot plots with error bars indicating the GMT with a 95% CI. The GMT values are shown on the axis X. Fold changes and *P* values of GMTs compared to WT by Omicron variants are shown at the top of each group. *P < 0.05, **P < 0.01, ***P < 0.001, ****P < 0.0001. Pie charts show the proportion of individuals within each group that had detectable neutralization against the indicated SARS-CoV-2 variants. All neutralization assays were conducted in biological duplicates.

We also detected nAbs of Omicron inpatients and found that booster doses could increase the breadth and magnitude of neutralization against WT and Omicron subvariants at the acute phase of Omicron infection, resulting in 49.4-fold and 3.7-fold enhancement of GMTs against WT and CH1.1 in hybrid-vaccinated inpatients compared to three-dose vaccinated inpatients (Fig. 5A; Table S5).

Although the neutralizing abilities in inpatients were comparable in survival cases compared to non-survival cases, the detection rates for WT (*P* = 0.22), B.1.1.529 (*P* = 0.05), BA.5 (*P* = 0.001), BF.7 (*P* = 0.02), and CH1.1 (*P* = 0.32) were significantly lower in the non-survival group (36.4%, 54.5%, 50%, and 72.7%, respectively) than in the survival group (64.3%, 96.4%, 82.1%, and 82.1%, respectively) (Fig. 5B; Table S6).

### Naturally infected individuals have a higher nasal mucosal IgA response

**Fig 6:**
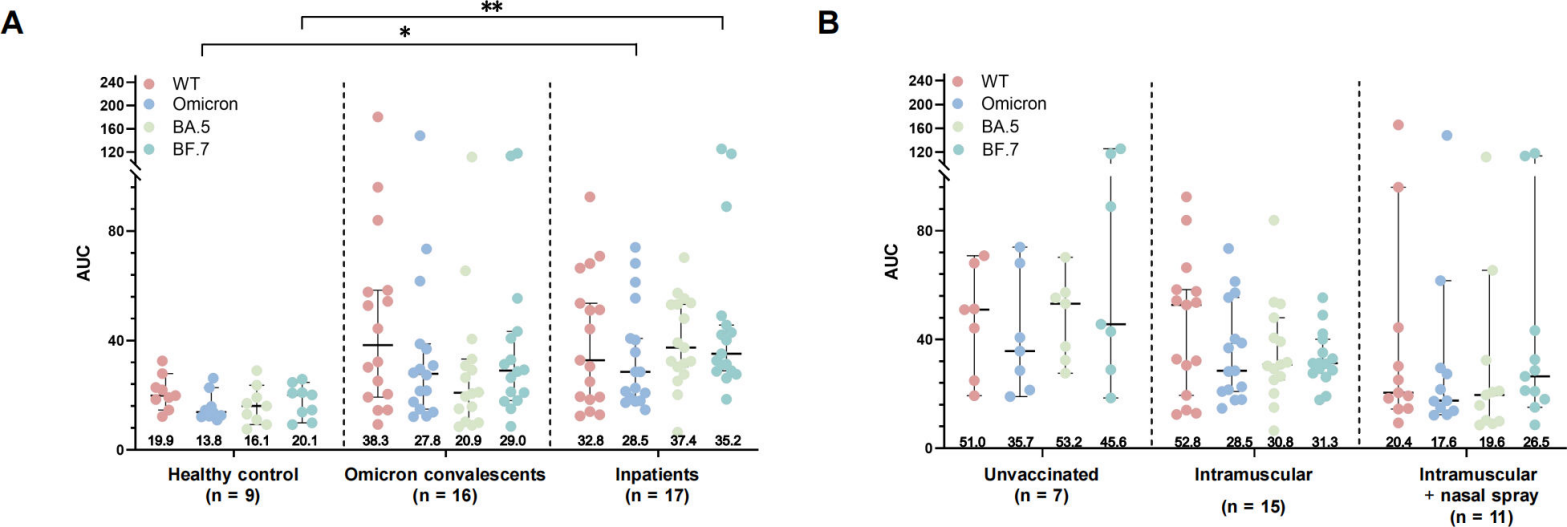
Nasal mucosal specific IgA levels against WT and Omicron subvariants in different subgroups. (**A**) The area under the curve (AUC) of the IgA levels was determined *via* ELISA assay against WT (pink dots), B.1.1.529 (blue dots), BA.5 (green dots), and BF.7 (dark green dots) in healthy donors, Omicron convalescents, and inpatients. (**B**) IgA levels against WT and Omicron variants in Omicron-infected individuals who have not been vaccinated (unvaccinated), have received three doses of inactivated vaccine (intramuscular), or have received three doses of inactivated vaccine and one dose of nasal spray vaccine (intramuscular + nasal spray). Data are presented as scatter dot plots with error bars indicating the geometric average IgA level with a 95% CI. The *P* values between different groups are shown at the top of the figure. *P < 0.05, **P < 0.01, ***P < 0.001, ****P < 0.0001.

We further characterized the SARS-CoV-2-specific nasal mucosal IgA in 42 participants, including 9 healthy donors, 16 Omicron convalescents, and 17 Omicron inpatients. The nasal mucosal IgA was detectable in all participants. Levels of IgA against WT, B.1.1.529, BA.5, and BF.7 in healthy participants (AUC 19.9, 13.8, 16.1, and 20.1, respectively) were lower than those in Omicron convalescents (AUC 38.3, 27.8, 20.9, and 29, respectively) and inpatients (AUC 32.8, 28.5, 37.4, and 35.2, respectively) (Fig. 6A). In addition, nasal spray vaccination did not induce higher nasal mucosal IgA responses than non-vaccination or intramuscular vaccination (Fig. 6B; Table S7). The data indicated that natural infection induced stronger nasal mucosal IgA levels against Omicron and WT than vaccination.

### Levels of serum nAbs and mucosal IgA were not related to cytokine levels or viral loads

We observed that nAbs against BF.7 (*P* < 0.05, r^2^ = 0.2328) and CH1.1 (*P* < 0.01, r^2^ = 0.3015) were weakly correlated with the interleukin 8 (IL-8) (Fig. S2F). Meanwhile, nAbs against WT (*P* < 0.001, r^2^ = 0.3863), BF.7 (*P* < 0.05, r^2^ = 0.2913), and CH1.1 (*P* < 0.01, r^2^ = 0.3325) were correlated with IL-12 (Fig. S2I). No correlations were observed between nAbs and other cytokines, including IL-1β, IL-2, IL-4, IL-5, IL-6, IL-10, IL-17, IFNα, IFNγ, and TNFα (all *P* > 0.05) (Fig. S2). Likewise, we did not find significant correlations between nasal mucosa IgA levels, cytokines, and cycle threshold (Ct) values (Fig. S3A, S3B, and S4).

## DISCUSSION

In the current study, we characterized Omicron-specific humoral and nasal mucosal immunity in various Chinese populations after the national wave of BA.5/BF.7 in early 2023. Natural infection elicited stronger humoral and mucosal activities against Omicron variants than a booster vaccination. A fourth dose revealed limited potential to improve nAbs against Omicron variants in both vaccinees and convalescents. However, they induced the most potent pan-Omicron neutralizing abilities in Omicron inpatients. No significant reduction of nAbs was observed during a 1-year follow-up, regardless of whether people experienced primary infection, re-infection, or further vaccination. The data indicated that further immunization may still be beneficial for those who are at higher risk of hospitalization, even though it may only slightly improve antibody responses in the general population.

Previous research has indicated that natural infection could elicit higher neutralizing activities than vaccination against both WT and Omicron variants (13, 14), in our study, we also identified that natural infections increased nAbs against WT and CH1.1 by 18.7–37.7 times and 1.3–3.7 times, respectively. In addition, the comparison of nAbs before and after a 1-year follow-up of the same person also confirmed the conclusion. We further demonstrated that BA.5/BF.7 breakthrough infection enhanced the neutralizing activities specifically against CH1.1 than delta breakthrough infection. Also, Bekliz et al. showed that Delta breakthrough infection generated a weaker neutralizing ability against BA.1 than Omicron breakthrough infection (15). However, Venice et al. found that Omicron breakthrough infections generate a slower rise in and lower levels of neutralizing antibodies against WT than Delta, indicating that Omicron breakthrough infections are less immunogenic than Delta (16). Various spike mutations in Omicron may cause different immune profiles compared to the other VOCs (17). In addition, affinity maturation caused by Omicron antigen exposure can markedly expand the breadth and efficiency of nAbs against SARS-CoV-2 infection (18).

The persistence of nAbs has emerged as a critical area of interest. Current understanding suggests that nAbs against VOCs typically decline after approximately 6 months (9, 19). Consequently, it has been proposed that the optimal interval for vaccine administration should be set at 6 months to maintain robust immunity (20). However, a recent study by Liew et al. demonstrated that the plasma-neutralizing titers against the Delta and BA.1 variants in COVID-19 inpatients remained stable over 12 months (21). This intriguing observation resonates with our results, underscores the complexity of the immune response, and highlights the need for ongoing research to refine vaccination strategies. As in our study, we found that the decline times of nAbs against Omicron variants were prolonged to nearly 1 year. Besides, continued waves of Omicron were not observed in mid-2023, as indicated in predicted models (22, 23), which partially supports our results that nAbs against Omicron variants may last a longer time than VOCs. More large-scale research is needed to identify the exact duration time of nAbs against Omicron variants.

Wang et al. reported weakened neutralizing responses against Omicron and its variants in participants who received a third or fourth dose of inactivated vaccine after BA.5/BF.7 breakthrough infection, compared to full vaccination (24). They assumed that repeated vaccination with an inactivated virus vaccine back-boosts previous memory and dampens the immune response to a new antigenically related but distinct viral strain (7, 24). However, we did not observe a significant decrease in nAbs against Omicron subvariants with increasing vaccination for various populations in different states. The controversial results may be due to the different antigenic epitopes of Omicron variants, as different strains of Omicron exhibited different responses to repeated vaccination (25). For example, a fourth dose further elevated the peak level of nAbs against SARS-CoV-2 and Omicron BA.2, but not BA.1, which had more NTD mutations (25). Larger-scale research is still needed to clarify the impact of additional vaccination on different subtypes of Omicron infection.

In our study, the nAbs were higher in severe inpatients as the increasing doses of vaccination at disease onset, and the detection rates of nAbs against Omicron variants were higher in the survival group compared to the non-survival group. Thus, we assume that further vaccination still has practical value for populations at high risk of hospitalization. Higher nAbs were known as a predictor of survival (26, 27). Studies also demonstrated that three doses of either mRNA or inactivated vaccine provide substantial additional protection against severe COVID-19 (28, 29). Besides, for individuals with poor response, continued vaccination may contribute to keeping peripheral nAb levels high, limiting infection, and potential transmission (30, 31).

Mucosal immunity has shown distinct protection activities against SARS-COV-2 (32, 33). Previous studies have shown the nasal booster strongly induced mucosal immunity as evidenced by the 21-fold increase in IgG and 11-fold increase in IgA against Omicron RBD in bronchoalveolar lavage fluid (BALF) (32), as well as Beta and Delta (33). We did not observe a robust elevation of nasal IgA levels after vaccination, but breakthrough infection did increase mucosal IgA levels. Harold et al. also showed that breakthrough infection, rather than mRNA and/or inactivated vaccines, produced higher IgA responses against BA.1, BA.2, and BA.4/5. Besides, additional vaccine doses did not boost the salivary IgA response against Omicron variants (34). Correlations between nasal swab Ct values and IgA titers were not observed either, although higher nasal S-RBD-specific antibody levels were associated with lower viral load (35).

Although we illustrated various immune responses against Omicron variants in different populations, we still have some limitations. First, our study is a relatively small cohort and we did not conduct further follow-up on Omicron inpatients, which may help us better to understand the role of humoral and mucosal immunity. Second, the currently most concerned Omicron variants, such as JN.1, were not included. Finally, despite conducting nucleic acid tests and inquiring about respiratory symptoms prior to sample collection, it is impossible to rule out asymptomatic infections among the healthy controls completely.

### Conclusion

Our data revealed that being infected with the Omicron variants induces a stronger and wider range of immune responses compared to vaccination or prior infection with other variants of SARS-CoV-2. While the fourth dose of an inactive vaccine had limited impact on boosting immunity against Omicron, continuing vaccination may still offer protection for vulnerable populations at high risk of hospitalization. We need to focus on longitudinal studies to track immune response durability for future research to clarify the mechanism of the vaccines.

## Data Availability

The data that support the findings of this study are available within the article and its supplementary materials. Raw data are available from the corresponding authors upon reasonable request.

## SUPPLEMENTAL MATERIAL

Fig. S1 spectrum.01117-24-s0001.tif Neutralizing antibody titers in Delta cohort.

Fig. S2 spectrum.01117-24-s0002.pdf NAbs showed little correlation with other cytokines.

Fig. S3 spectrum.01117-24-s0003.pdf IgA showed no correlation with nAbs and Ct values.

Fig. S4 spectrum.01117-24-s0004.pdf No correlation between IgA and cytokines.

Supplemental material spectrum.01117-24-s0005.docx Supplementary figure legends.

Supplemental tables spectrum.01117-24-s0006.doc Tables S1 to S7.

ASM does not own the copyrights to Supplemental Material that may be linked to, or accessed through, an article. The authors have granted ASM a non-exclusive, world-wide license to publish the Supplemental Material files. Please contact the corresponding author directly for reuse.

## References

[1] WHO. 2023. Statement-on-the-fourteenth-meeting-of-the-international-health-regulations-(2005)-emergency-committee-regarding-the-coronavirus-disease-(Covid-19)-pandemic. Available from: https://www.who.int/news/item/30-01-2023-statement-on-the-fourteenth-meeting-of-the-international-health-regulations-(2005)-emergency-committee-regarding-the-coronavirus-disease-(covid-19)-pandemic

[2] Yuan S, Ye Z-W, Liang R, Tang K, Zhang AJ, Lu G, Ong CP, Man Poon VK, Chan CC-S, Mok BW-Y, et al.. 2022. Pathogenicity, transmissibility, and fitness of SARS-CoV-2 Omicron in Syrian hamsters. Science 377:428–433. doi:10.1126/science.abn8939 35737809

[3] Shi G, Li T, Lai KK, Johnson RF, Yewdell JW, Compton AA. 2024. Omicron Spike confers enhanced infectivity and interferon resistance to SARS-CoV-2 in human nasal tissue. Nat Commun 15:889. doi:10.1038/s41467-024-45075-8 38291024 PMC10828397

[4] National Health Commission of the People’s Republic of China. 2022. Text transcript of the press conference of the State Council’s joint prevention and control mechanism on November 12, 2022. Available from: http://www.nhc.gov.cn/xcs/s3574/202211/b20c7ccccf4842af874a7781392311e5.shtml

[5] Callaway E. 2022. COVID “variant soup” is making winter surges hard to predict. Nature New Biol 611:213–214. doi:10.1038/d41586-022-03445-6 36307585

[6] Zhu F, Zhuang C, Chu K, Zhang L, Zhao H, Huang S, Su Y, Lin H, Yang C, Jiang H, et al.. 2022. Safety and immunogenicity of a live-attenuated influenza virus vector-based intranasal SARS-CoV-2 vaccine in adults: randomised, double-blind, placebo-controlled, phase 1 and 2 trials. Lancet Respir Med 10:749–760. doi:10.1016/S2213-2600(22)00131-X 35644168 PMC9135375

[7] Gao B, He L, Bao Y, Chen Y, Lu G, Zhang Y, Xu Y, Su B, Xu J, Wang Y, Yeap L-S. 2023. Repeated vaccination of inactivated SARS-CoV-2 vaccine dampens neutralizing antibodies against Omicron variants in breakthrough infection. Cell Res 33:258–261. doi:10.1038/s41422-023-00781-8 36697703 PMC9875169

[8] Yu X, Wei D, Xu W, Li Y, Li X, Zhang X, Qu J, Yang Z, Chen E. 2022. Reduced sensitivity of SARS-CoV-2 Omicron variant to antibody neutralization elicited by booster vaccination. Cell Discov 8:4. doi:10.1038/s41421-022-00375-5 35034952 PMC8761745

[9] Li J, Wu J, Long Q, Wu Y, Hu X, He Y, Jiang M, Li J, Zhao L, Yang S, Chen X, Wang M, Zheng J, Wu F, Wu R, Ren L, Bu L, Wang H, Li K, Fu L, Zhang G, Zheng Y, Gao Z. 2022. Comprehensive humoral and cellular immune responses to SARS-CoV-2 variants in diverse Chinese population. Research (Lambertville) 2022:9873831. doi:10.34133/2022/9873831 PMC9275105 35935138

[10] Nie J, Li Q, Wu J, Zhao C, Hao H, Liu H, Zhang L, Nie L, Qin H, Wang M, Lu Q, Li X, Sun Q, Liu J, Fan C, Huang W, Xu M, Wang Y. 2020. Quantification of SARS-CoV-2 neutralizing antibody by a pseudotyped virus-based assay. Nat Protoc 15:3699–3715. doi:10.1038/s41596-020-0394-5 32978602

[11] Di Genova C, Sampson A, Scott S, Cantoni D, Mayora-Neto M, Bentley E, Mattiuzzo G, Wright E, Derveni M, Auld B, Ferrara BT, Harrison D, Said M, Selim A, Thompson E, Thompson C, Carnell G, Temperton N. 2021. Production, titration, neutralisation, storage and lyophilisation of severe acute respiratory syndrome Coronavirus 2 (SARS-CoV-2) lentiviral pseudotypes. Bio Protoc 11:e4236. doi:10.21769/BioProtoc.4236 PMC8595416 34859134

[12] Chinese Center for Disease Control and Prevention. 2023. Available from: https://www.chinacdc.cn/jkzt/crb/zl/szkb_11803/jszl_2275/202302/t20230201_263573.html 10.46234/ccdcw2020.084 PMC8392945 34594650

[13] Holmer HK, Mackey K, Fiordalisi CV, Helfand M. 2023. Major update 2: antibody response and risk for reinfection after SARS-CoV-2 infection-final update of a living, rapid review. Ann Intern Med 176:85–91. doi:10.7326/M22-1745 36442059 PMC9707440

[14] Michlmayr D, Hansen CH, Gubbels SM, Valentiner-Branth P, Bager P, Obel N, Drewes B, Møller CH, Møller FT, Legarth R, Mølbak K, Ethelberg S. 2022. Observed protection against SARS-CoV-2 reinfection following a primary infection: a Danish cohort study among unvaccinated using two years of nationwide PCR-test data. Lancet Reg Health Eur 20:100452. doi:10.1016/j.lanepe.2022.100452 35791335 PMC9245510

[15] Bekliz M, Adea K, Vetter P, Eberhardt CS, Hosszu-Fellous K, Vu D-L, Puhach O, Essaidi-Laziosi M, Waldvogel-Abramowski S, Stephan C, L’Huillier AG, Siegrist C-A, Didierlaurent AM, Kaiser L, Meyer B, Eckerle I. 2022. Neutralization capacity of antibodies elicited through homologous or heterologous infection or vaccination against SARS-CoV-2 VOCs. Nat Commun 13:3840. doi:10.1038/s41467-022-31556-1 35787633 PMC9253337

[16] Servellita V, Syed AM, Morris MK, Brazer N, Saldhi P, Garcia-Knight M, Sreekumar B, Khalid MM, Ciling A, Chen P-Y, Kumar GR, Gliwa AS, Nguyen J, Sotomayor-Gonzalez A, Zhang Y, Frias E, Prostko J, Hackett J Jr, Andino R, Wadford DA, Hanson C, Doudna J, Ott M, Chiu CY. 2022. Neutralizing immunity in vaccine breakthrough infections from the SARS-CoV-2 Omicron and Delta variants. Cell 185:1539–1548. doi:10.1016/j.cell.2022.03.019 35429436 PMC8930394

[17] Cui Z, Liu P, Wang N, Wang L, Fan K, Zhu Q, Wang K, Chen R, Feng R, Jia Z, Yang M, Xu G, Zhu B, Fu W, Chu T, Feng L, Wang Y, Pei X, Yang P, Xie XS, Cao L, Cao Y, Wang X. 2022. Structural and functional characterizations of infectivity and immune evasion of SARS-CoV-2 Omicron. Cell 185:860–871. doi:10.1016/j.cell.2022.01.019 35120603 PMC8786603

[18] Wratil PR, Stern M, Priller A, Willmann A, Almanzar G, Vogel E, Feuerherd M, Cheng C-C, Yazici S, Christa C, et al.. 2022. Three exposures to the spike protein of SARS-CoV-2 by either infection or vaccination elicit superior neutralizing immunity to all variants of concern. Nat Med 28:496–503. doi:10.1038/s41591-022-01715-4 35090165

[19] Bar-On YM, Goldberg Y, Mandel M, Bodenheimer O, Freedman L, Kalkstein N, Mizrahi B, Alroy-Preis S, Ash N, Milo R, Huppert A. 2021. Protection of BNT162b2 vaccine booster against Covid-19 in Israel. N Engl J Med 385:1393–1400. doi:10.1056/NEJMoa2114255 34525275 PMC8461568

[20] Levin EG, Lustig Y, Cohen C, Fluss R, Indenbaum V, Amit S, Doolman R, Asraf K, Mendelson E, Ziv A, Rubin C, Freedman L, Kreiss Y, Regev-Yochay G. 2021. Waning immune humoral response to BNT162b2 Covid-19 vaccine over 6 months. N Engl J Med 385:e84. doi:10.1056/NEJMoa2114583 34614326 PMC8522797

[21] Liew F, Talwar S, Cross A, Willett BJ, Scott S, Logan N, Siggins MK, Swieboda D, Sidhu JK, Efstathiou C, et al.. 2023. SARS-CoV-2-specific nasal IgA wanes 9 months after hospitalisation with COVID-19 and is not induced by subsequent vaccination. EBioMedicine 87:104402. doi:10.1016/j.ebiom.2022.104402 36543718 PMC9762734

[22] Mallapaty S. 2022. China COVID wave could kill one million people, models predict. Nature. doi:10.1038/d41586-022-04502-w 36536129

[23] Wang S-T, Wu Y-P, Li L, Li Y, Sun G-Q. 2023. Forecast for peak infections in the second wave of the Omicron after the adjustment of zero-COVID policy in the mainland of China. Infect Dis Model 8:562–573. doi:10.1016/j.idm.2023.05.007 37305609 PMC10251123

[24] Wang H, Xue Q, Zhang H, Yuan G, Wang X, Sheng K, Li C, Cai J, Sun Y, Zhao J, et al.. 2023. Neutralization against Omicron subvariants after BA.5/BF.7 breakthrough infection weakened as virus evolution and aging despite repeated prototype-based vaccination. Emerg Microbes Infect 12:2249121. doi:10.1080/22221751.2023.2249121 37668156 PMC10524800

[25] Wang J, Deng C, Liu M, Liu Y, Li L, Huang Z, Shang L, Jiang J, Li Y, Mo R, Zhang H, Liu M, Peng S, Xiao H. 2022. A fourth dose of the inactivated SARS-CoV-2 vaccine redistributes humoral immunity to the N-terminal domain. Nat Commun 13:6866. doi:10.1038/s41467-022-34633-7 36369243 PMC9651894

[26] Röltgen K, Powell AE, Wirz OF, Stevens BA, Hogan CA, Najeeb J, Hunter M, Wang H, Sahoo MK, Huang C, et al.. 2020. Defining the features and duration of antibody responses to SARS-CoV-2 infection associated with disease severity and outcome. Sci Immunol 5:eabe0240. doi:10.1126/sciimmunol.abe0240 33288645 PMC7857392

[27] Garcia-Beltran WF, Lam EC, Astudillo MG, Yang D, Miller TE, Feldman J, Hauser BM, Caradonna TM, Clayton KL, Nitido AD, Murali MR, Alter G, Charles RC, Dighe A, Branda JA, Lennerz JK, Lingwood D, Schmidt AG, Iafrate AJ, Balazs AB. 2021. COVID-19-neutralizing antibodies predict disease severity and survival. Cell 184:476–488. doi:10.1016/j.cell.2020.12.015 33412089 PMC7837114

[28] Kundi M. 2023. Vaccine effectiveness against Delta and Omicron variants of SARS-CoV-2. BMJ 381:1111. doi:10.1136/bmj.p1111 37220942

[29] McMenamin ME, Nealon J, Lin Y, Wong JY, Cheung JK, Lau EHY, Wu P, Leung GM, Cowling BJ. 2022. Vaccine effectiveness of one, two, and three doses of BNT162b2 and CoronaVac against COVID-19 in Hong Kong: a population-based observational study. Lancet Infect Dis 22:1435–1443. doi:10.1016/S1473-3099(22)00345-0 35850128 PMC9286709

[30] Lake DF, Roeder AJ, Gonzalez-Moa MJ, Koehler M, Kaleta E, Jasbi P, Vanderhoof J, McKechnie D, Forman J, Edwards BA, Seit-Nebi A, Svarovsky S. 2022. Third COVID-19 vaccine dose boosts neutralizing antibodies in poor responders. Commun Med (Lond) 2:85. doi:10.1038/s43856-022-00151-2 35832309 PMC9273613

[31] Centers for Disease Control and Prevention. 2024. Interim effectiveness of updated 2023–2024 (Monovalent XBB.1.5) COVID-19 vaccines against COVID-19–associated hospitalization among adults aged ≥18 years with immunocompromising conditions — VISION network, September 2023–February 2024. Available from: https://www.cdc.gov/mmwr/volumes/73/wr/mm7312a5.htm?s_cid=mm7312a5_w 10.15585/mmwr.mm7312a5 PMC10986819 38547037

[32] Lam J-Y, Ng Y-Y, Yuen C-K, Wong W-M, Yuen K-Y, Kok K-H. 2022. A nasal Omicron vaccine booster elicits potent neutralizing antibody response against emerging SARS-CoV-2 variants. Emerg Microbes Infect 11:964–967. doi:10.1080/22221751.2022.2053365 35275039 PMC8973333

[33] Wang Q, Yang C, Yin L, Sun J, Wang W, Li H, Zhang Z, Chen S, Liu B, Liu Z, Shi L, Liu X, Guan S, Wang C, Qu L, Feng Y, Niu X, Feng L, Zhao J, Li P, Chen L, Zhong N. 2023. Intranasal booster using an Omicron vaccine confers broad mucosal and systemic immunity against SARS-CoV-2 variants. Signal Transduct Target Ther 8:167. doi:10.1038/s41392-023-01423-6 37069171 PMC10106878

[34] Marcotte H, Cao Y, Zuo F, Simonelli L, Sammartino JC, Pedotti M, Sun R, Cassaniti I, Hagbom M, Piralla A, et al.. 2024. Conversion of monoclonal IgG to dimeric and secretory IgA restores neutralizing ability and prevents infection of Omicron lineages. Proc Natl Acad Sci U S A 121:e2315354120. doi:10.1073/pnas.2315354120 38194459 PMC10801922

[35] Fröberg J, Gillard J, Philipsen R, Lanke K, Rust J, van Tuijl D, Teelen K, Bousema T, Simonetti E, van der Gaast-de Jongh CE, Bos M, van Kuppeveld FJ, Bosch B-J, Nabuurs-Franssen M, van der Geest-Blankert N, van Daal C, Huynen MA, de Jonge MI, Diavatopoulos DA. 2021. SARS-CoV-2 mucosal antibody development and persistence and their relation to viral load and COVID-19 symptoms. Nat Commun 12:5621. doi:10.1038/s41467-021-25949-x 34556667 PMC8460778

