# Supplementary figures and images for: Heterogeneity of SARS-CoV-2 immune responses after the nationwide Omicron wave in China
Source: Microbiol Spectr. 2024 Sep 17;12(11):e01117-24. doi: 10.1128/spectrum.01117-24 (PMC11536994; doi:10.1128/spectrum.01117-24)

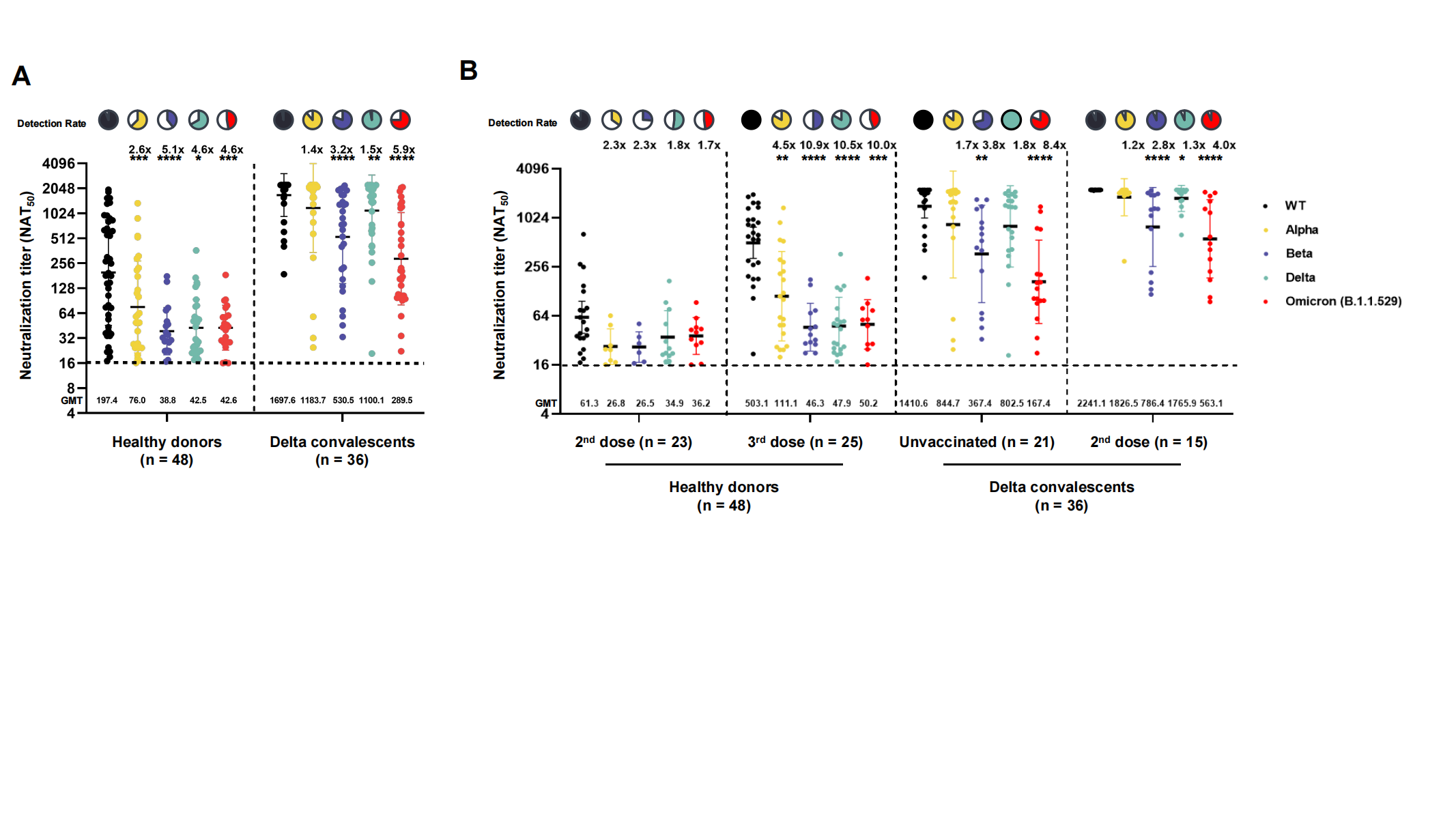

Supplement: Fig. S1 — Neutralizing antibody titers in Delta cohort. [file spectrum.01117-24-s0001.tif]

**A****IL-1 $\beta$** 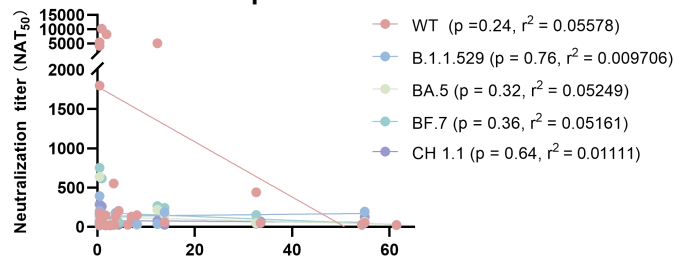**B****IL-2**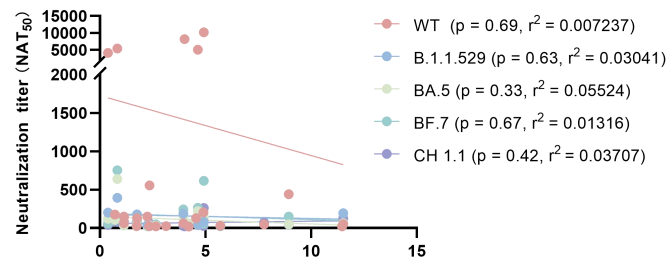**C****IL-4**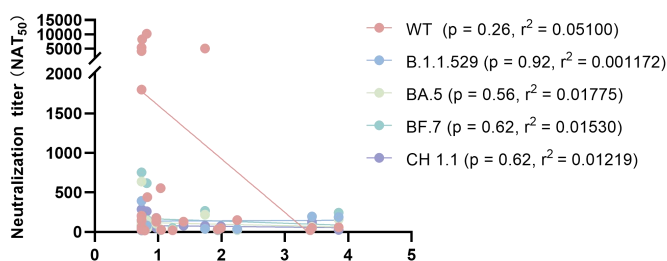**D****IL-5**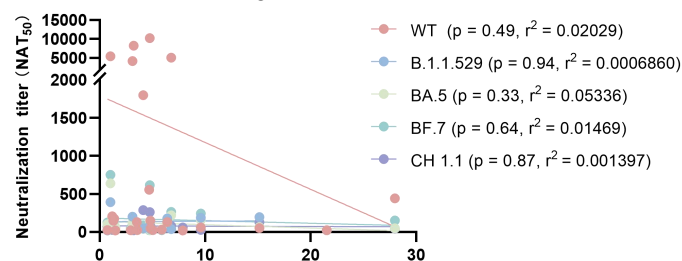**E****IL-6**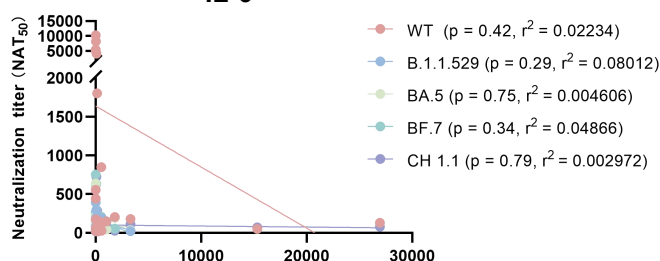**F****IL-8**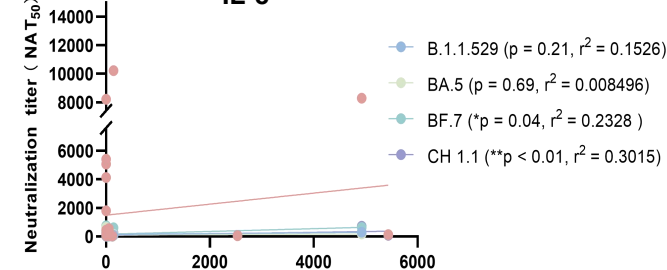**G****IL-10**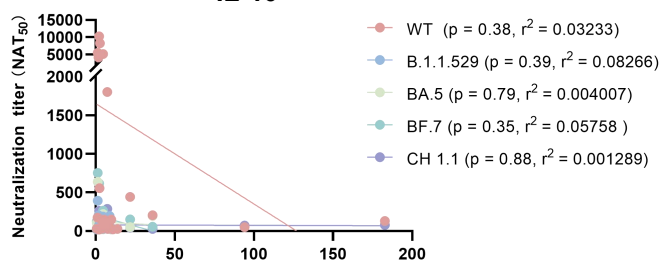**H****IL-17**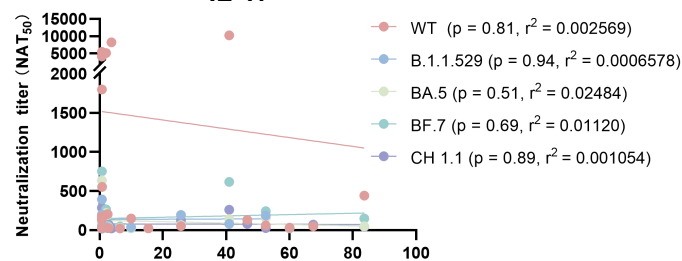**I****IL-12**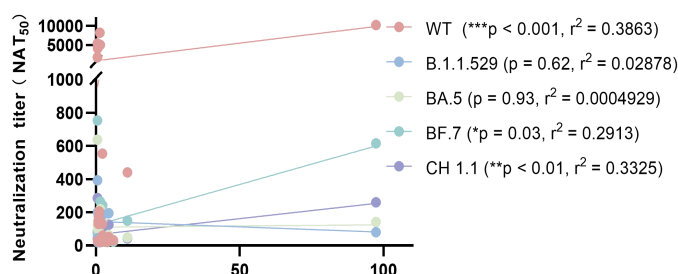**J****IFN $\alpha$** 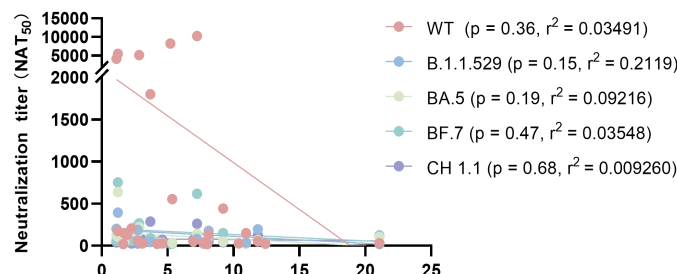**K****IFN $\gamma$** 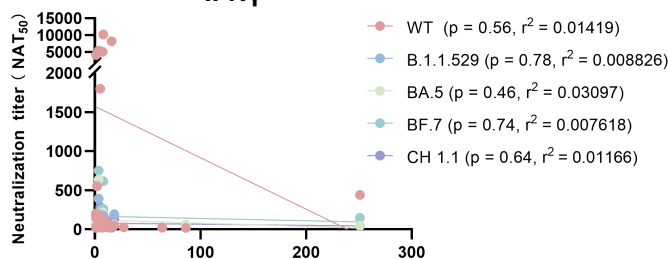**L****TNF $\alpha$** 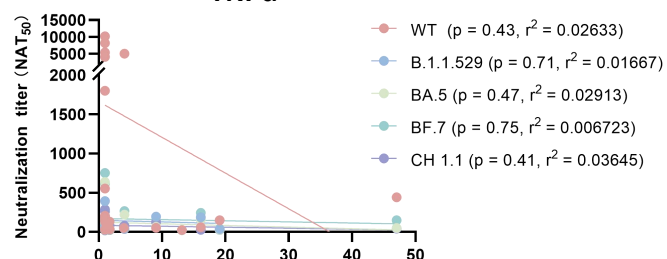

Supplement: Fig. S2 — NAbs showed little correlation with other cytokines. [file spectrum.01117-24-s0002.pdf]

**A**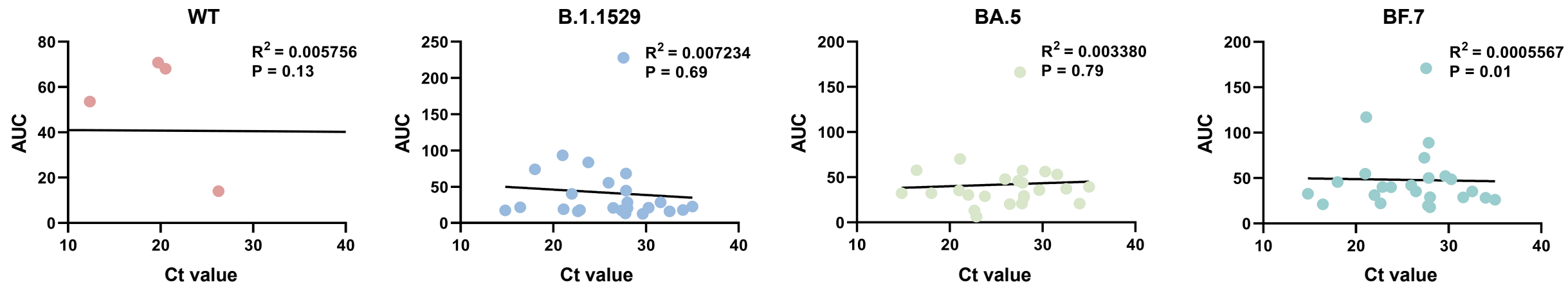**B**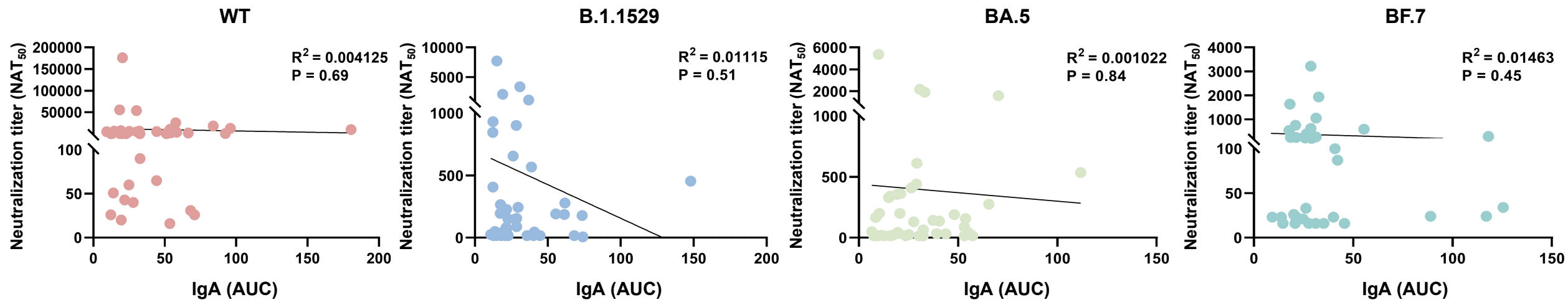

Supplement: Fig. S3 — IgA showed no correlation with nAbs and Ct values. [file spectrum.01117-24-s0003.pdf]

**A**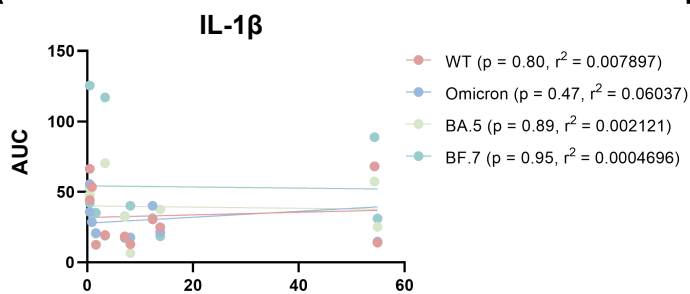**B**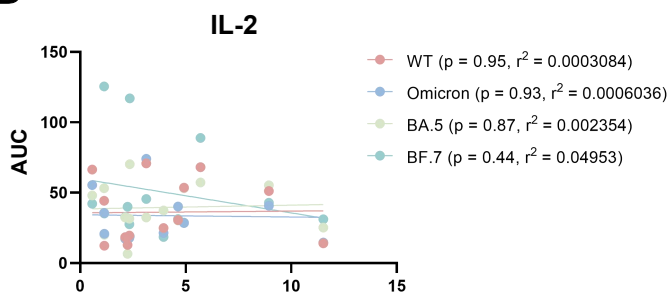**C**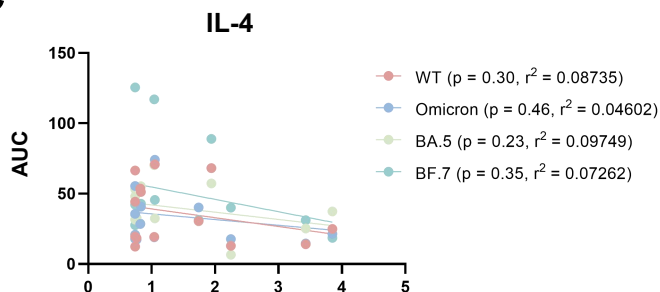**D**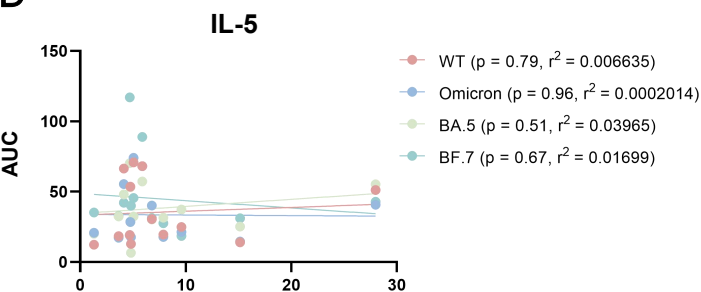**E**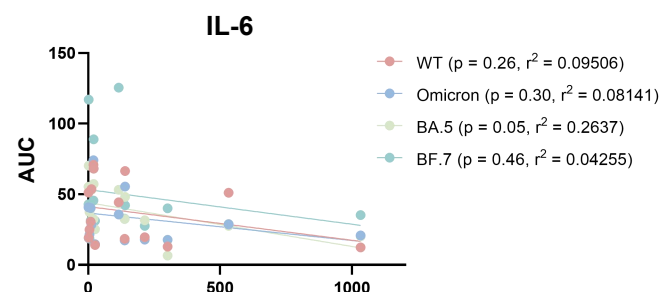**F**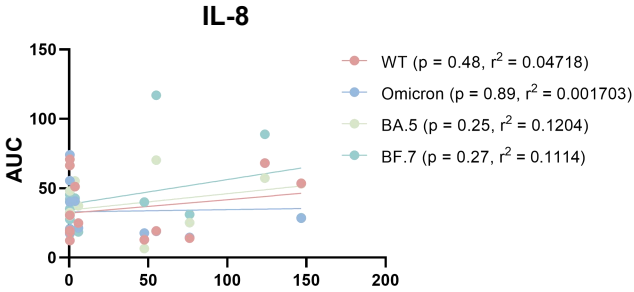**G**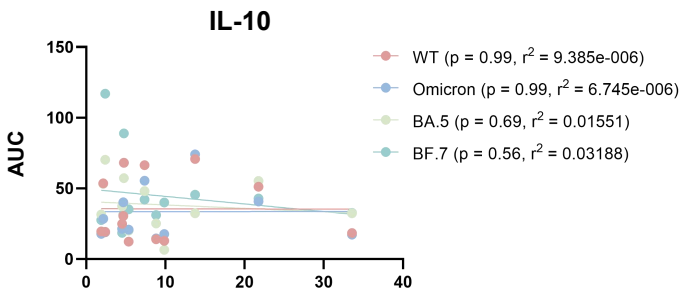**H**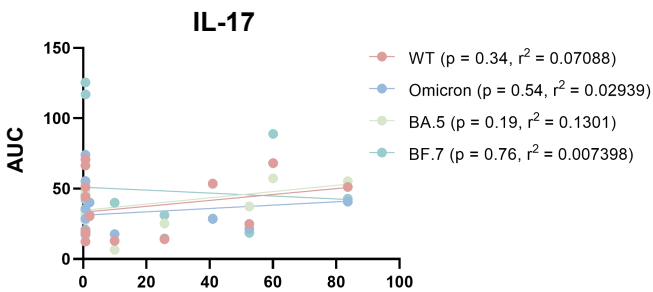**I**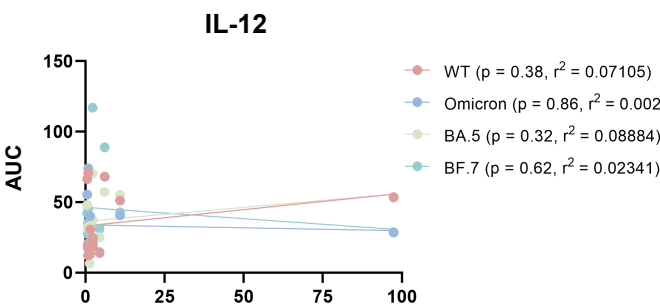**J**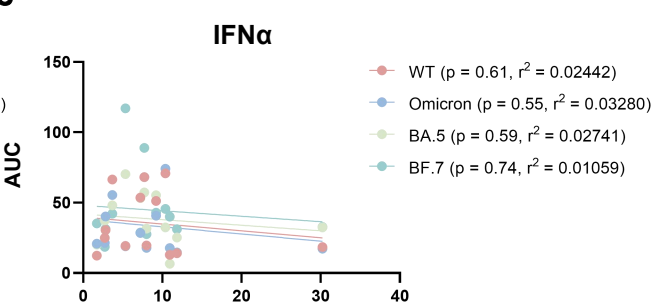**K**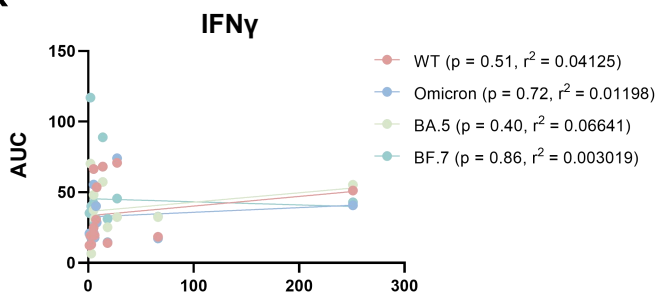**L**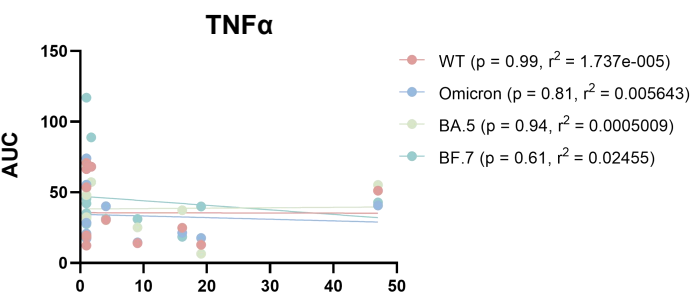

Supplement: Fig. S4 — No correlation between IgA and cytokines. [file spectrum.01117-24-s0004.pdf]
