# Supplementary material for: Heterogeneity of SARS-CoV-2 immune responses after the nationwide Omicron wave in China
Source: Microbiol Spectr. 2024 Sep 17;12(11):e01117-24. doi: 10.1128/spectrum.01117-24 (PMC11536994; doi:10.1128/spectrum.01117-24)
Supplement: Supplemental material — Supplementary figure legends. [file spectrum.01117-24-s0005.docx]

**Supplementary Figure S1. Neutralizing antibody titers in Delta cohort. (A)** The 50% neutralization titers (NAT50) were determined via VSV pseudovirus neutralization assay against WT (black dots), and variants of concerns (VOCs, Alpha (yellow dots), Beta (purple dots), Delta (green dots), and Omicron B.1.1.529 (red dots)) in healthy donors and Delta convalescents. **(B)** The nAbs against WT and VOCs in healthy donors and Delta convalescents with different vaccination backgrounds. Data are presented as scatter dot plots with error bars indicating the GMT with a 95% CI. The GMT values are shown on the axis X. Fold-changes and p values of GMTs compared to WT by VOCs are shown at the top of each group. * p < 0.05, ** p < 0.01, *** p < 0.001, **** p < 0.0001. Pie charts show the proportion of individuals within each group that had detectable neutralization against the indicated SARS-CoV-2 variants. All neutralization assays were conducted in biological duplicates.

**Supplementary Figure S2. NAbs showed little correlation with other cytokines.** The correlation between nAbs and cytokines including IL-1β **(A)**, IL-2 **(B)**, IL-4 **(C)**, IL-5 **(D)**, IL-6 **(E)**, IL-8 **(F)**, IL-10 **(G),** IL-17 **(H)**, IL-12 **(I)**, IFNα **(J)**, IFNγ**(K)**, and TNFα **(L)**. Different colored dots and lines represent the nAbs and correlation linear of WT (pink), B.1.1.529 (blue), BA.5 (green), BF.7 (dark green), and CH1.1 (purple). * p < 0.05, * * p < 0.01, * * * p < 0.001.

**Supplementary Figure S3**. **IgA showed no correlation with nAbs and Cycle threshold (Ct) values.** The correlation between nasal mucosal-specific IgA levels, Ct values **(A)**, and nAbs **(B)**. Different colored dots and lines represent the IgA and correlation linear of WT (pink), B.1.1.529 (blue), BA.5 (green), and BF.7 (dark green). * p < 0.05, * * p < 0.01, * * * p < 0.001.

**Supplementary Figure S4. No correlation between IgA and cytokines.** The correlation between nasal mucosal-specific IgA levels and cytokines including IL-1β **(A)**, IL-2 **(B)**, IL-4 **(C)**, IL-5 **(D)**, IL-6 **(E)**, IL-8 **(F)**, IL-10 **(G)**, IL-17 **(H),** IL-12 **(I),** IFNα **(J)**, IFNγ **(K)**, and TNFα **(L)**. Different colored dots and lines represent the IgA and correlation linear of WT (pink), B.1.1.529 (blue), BA.5 (green), and BF.7 (dark green). * p < 0.05, * * p < 0.01, * * * p < 0.001.
