# Supplementary material for: Heterogeneity of SARS-CoV-2 immune responses after the nationwide Omicron wave in China
Source: Microbiol Spectr. 2024 Sep 17;12(11):e01117-24. doi: 10.1128/spectrum.01117-24 (PMC11536994; doi:10.1128/spectrum.01117-24)
Supplement: Supplemental tables — Tables S1 to S7. [file spectrum.01117-24-s0006.doc]

**Supplementary Table S1. GMT values with 95% CI in different subgroups.**

|  | | **WT** | **Alpha** | **Beta** | **Delta** | **B.1.1.529** | **BA.5** | **BF.7** | **CH1.1** |
| --- | --- | --- | --- | --- | --- | --- | --- | --- | --- |
| **All participants** | | 819.5  (549.3-1222.4) | 313.5  (195.5-502.7) | 188.4  (115.5-307.1) | 232.6  (147.6-366.7) | 177.7  (132.4-238.5) | 131.5  (102.5-168.6) | 121.8  (94.7-156.5) | 65.7  (53-81.6) |
| **Delta convalescent (2021.12)**  **(n = 36)** | **All convalescents** | 1697.6  (1383-2083) | 1183.7  (759.2-1846) | 530.5  (324.2-868.1) | 1100.1  (782.6-1546) | 289.5  (180.9-463.3) | - | - | - |
|  | **Unvaccinated**  **(n = 21)** | 1410.6  (1018-1955) | 844.7  (398.5-1791) | 367.4  (171.5-787.2) | 802.5  (475.7-1354) | 167.4  (91.4-306.5) | - | - | - |
|  | **2^nd^ dose**  **(n = 15)** | 2241.1  (2235-2248) | 1826.5  (1350-2472) | 786.4  (412.5-1499) | 1765.9  (1426-2187) | 563.1  (297.8-1065) | - | - | - |
| **Healthy donors (2021.12)**  **(n = 48)** | **All healthy donors** | 197.4  (93.9-263.8) | 76  (36.1-118.8) | 38.8  (26.2-61.8) | 42.5  (29.6-57.6) | 42.6  (31.3-59) | - | - | - |
|  | **2^nd^ dose**  **(n = 23)** | 61.3  (39-96.3) | 26.8  (17.6-40.7) | 26.5  (16.8-41.6) | 34.9  (21.6-56.3) | 36.2  (25.6-51.3) | - | - | - |
|  | **3^rd^ dose**  **(n = 25)** | 503.1  (322.9-784) | 111.1  (63.5-194.4) | 46.3  (30.7-63.6) | 47.9  (32.8-70) | 50.2  (31.4-80.2) | - | - | - |
| **Omicron convalescents**  **(2023)**  **(n = 19^a^)** | **All Omicron convalescents** | 11364.8  (6503.1-19861.3) | - | - | - | 513.5  (277.9-948.8) | 528.2  (331.3-842.2) | 409.5  (245-684.5) | 105.9  (62.4-180) |
|  | **3^rd^ dose**  **(n = 7)** | 10935.4  (2743.9-43581.8) | - | - | - | 410.5  (65.6-2570.7) | 966.1  (308.7-3023.1) | 680  (207.2-2231.5) | 126.7  (30.2-531.9) |
|  | **Hybrid vaccination^b^**  **(n = 11)** | 12288  (6211.6-24308.7) | - | - | - | 550.6  (349.9-866.3) | 375.5  (249.8-564.5) | 320.7  (180.3-570.4) | 65.1  (60.3-161.9) |
| **Omicron inpatients**  **(2023)**  **(n = 50)** | **All Omicron inpatients** | 292.2  (137.7-620) | - | - | - | 141.8  (81.8-245.9) | 93.3  (64.6-134.7) | 114.4  (78.6-193.9) | 77.6  (55.2-109.2) |
|  | **Survival**  **(n = 28)** | 337.8  (128.5-888.1) | - | - | - | 134.6  (68.6-264) | 85.4  (56.4-129.3) | 100.2  (66.5-191.8) | 93.5  (56.6-154.5) |
|  | **Non-Survival**  **(n = 22)** | 237.9  (64.4-878.7) | - | - | - | 159.6  (46.8-544.2) | 113.9  (48.8-265.5) | 150.9  (55.4-411) | 59.4  (38.1-92.6) |
|  | **Unvaccinated**  **(n = 26)** | 108.7  (56.3-209.7) | - | - | - | 76.5  (33.2-176.1) | 79.7  (52.7-120.5) | 79.9  (50.3-127) | 42.8  (33.1-55.4) |
|  | **1^st^-2^nd^ dose**  **(n = 13)** | 217.5  (49.7-952.3) | - | - | - | 82.5  (37.7-180.2) | 77  (37.6-157.6) | 88.4  (34.4-227.5) | 75  (51-110.2) |
|  | **3^rd^ dose**  **(n = 8)** | 1034.3  (112.4-9519.4) | - | - | - | 217.9  (63.1-753) | 76.1  (30.1-192.4) | 119.6  (28.6-500.6) | 140.8  (49-404.8) |
|  | **Hybrid vaccination**  **(n = 3)** | 51112.5  (617.1-4233702) | - | - | - | 1146.7  (62-21223.2) | 796.4  (29.3-21643.8) | 1486.6  (224.9-9827.7) | 527.7  (18.8-14811.6) |
| **Healthy donors^c^**  **(2023)**  **(n = 40)** | **All healthy donors** | 301.2  (177.2-512.2) | - | - | - | 59.1  (41.8-83.4) | 55.4  (40-76.6) | 45.8  (36.1-58) | 28.4  (22.3-36.1) |
|  | **2^nd^ dose**  **(n = 13)** | 66.2  (43.3-101.1) | - | - | - | 41.7  (28.8-60.4) | 58.3  (34.2-99.6) | 47.6  (35.1-64.8) | 20  (11.3-35.4) |
|  | **3^rd^ dose**  **(n = 12)** | 1069.3  (541.6-2111) | - | - | - | 71  (37.2-135.2) | 46.8  (30.9-70.9) | 46.4  (28.9-74.6) | 37.4  (24.9-56.4) |
|  | **Hybrid vaccination**  **(n = 15)** | 406.3  (162.2-1018) | - | - | - | 71.1  (31.4-161.2) | 63.1  (23.6-168.7) | 43.5  (24.8-76.3) | 23.7  (17 -33.1) |

^a^ One person received two doses of the vaccine.

^b^ Three doses of inactivated vaccine and additional nasal spray vaccine.

^c^ Including 20 healthy serum samples from the follow-up cohort established at the end of 2021 and 10 healthy serum samples after 1 year of follow-up.

**Supplementary Table S2. Fold changes and p values between different infection backgrounds.**

|  |  | **Fold change** | **p value** |
| --- | --- | --- | --- |
| **Delta convalescents / Healthy donors** | **WT** | 21.3 | 0.002 |
|  | **B.1.1.529** | 3.5 | 0.33 |
|  | **BA.5** | 5.4 | 0.02 |
|  | **BF.7** | 6.1 | 0.01 |
|  | **CH1.1** | 1.3 | > 0.99 |
| **Omicron convalescents / Healthy donors** | **WT** | 37.7 | < 0.0001 |
|  | **B.1.1.529** | 8.7 | < 0.0001 |
|  | **BA.5** | 9.5 | < 0.0001 |
|  | **BF.7** | 8.9 | < 0.0001 |
|  | **CH1.1** | 3.7 | 0.0002 |
| **Delta + Omicron convalescents / Healthy donors** | **WT** | 18.7 | 0.004 |
|  | **B.1.1.529** | 9.6 | 0.002 |
|  | **BA.5** | 6.6 | 0.006 |
|  | **BF.7** | 7.7 | 0.0008 |
|  | **CH1.1** | 3.6 | 0.04 |
| **Omicron convalescents / Delta convalescents** | **WT** | 1.8 | > 0.99 |
|  | **B.1.1.529** | 2.5 | 0.97 |
|  | **BA.5** | 1.8 | > 0.99 |
|  | **BF.7** | 1.5 | > 0.99 |
|  | **CH1.1** | 2.8 | 0.92 |
| **Delta + Omicron convalescents / Delta convalescents** | **WT** | 0.9 | > 0.99 |
|  | **B.1.1.529** | 2.8 | > 0.99 |
|  | **BA.5** | 1.2 | > 0.99 |
|  | **BF.7** | 1.3 | > 0.99 |
|  | **CH1.1** | 2.7 | > 0.99 |
| **Delta + Omicron convalescents / Omicron convalescents** | **WT** | 0.5 | > 0.99 |
|  | **B.1.1.529** | 1.1 | > 0.99 |
|  | **BA.5** | 0.7 | > 0.99 |
|  | **BF.7** | 0.9 | > 0.99 |
|  | **CH1.1** | 1 | > 0.99 |

**Supplementary Table S3. Fold changes and p values between different vaccination backgrounds.**

|  |  | **Fold change** | **p value** |
| --- | --- | --- | --- |
| **3^rd^ dose (healthy donors) / 2^nd^ dose (healthy donors)** | **WT** | 16.2 | 0.02 |
|  | **B.1.1.529** | 1.7 | > 0.99 |
|  | **BA.5** | 0.8 | > 0.99 |
|  | **BF.7** | 1 | > 0.99 |
|  | **CH1.1** | 1.9 | > 0.99 |
| **Hybrid**  **vaccination (healthy donors) / 2^nd^ dose (healthy donors)** | **WT** | 6.1 | < 0.0001 |
|  | **B.1.1.529** | 1.7 | > 0.99 |
|  | **BA.5** | 1.1 | > 0.99 |
|  | **BF.7** | 0.9 | > 0.99 |
|  | **CH1.1** | 1.2 | > 0.99 |
| **Hybrid**  **vaccination (healthy donors) / 3^rd^ dose (healthy donors)** | **WT** | 0.4 | > 0.99 |
|  | **B.1.1.529** | 1 | > 0.99 |
|  | **BA.5** | 1.3 | > 0.99 |
|  | **BF.7** | 0.9 | > 0.99 |
|  | **CH1.1** | 0.6 | > 0.99 |
| **Hybrid**  **vaccination (Omicron convalescents) / 3^rd^ dose (Omicron convalescents)** | **WT** | 1.1 | > 0.99 |
|  | **B.1.1.529** | 1.3 | > 0.99 |
|  | **BA.5** | 0.4 | > 0.99 |
|  | **BF.7** | 0.5 | > 0.99 |
|  | **CH1.1** | 0.5 | > 0.99 |
| **3^rd^ dose (Omicron convalescents) / 3^rd^ dose (healthy donors)** | **WT** | 10.2 | 0.39 |
|  | **B.1.1.529** | 5.8 | 0.74 |
|  | **BA.5** | 20.6 | 0.0008 |
|  | **BF.7** | 14.7 | 0.003 |
|  | **CH1.1** | 3.4 | 0.7 |
| **Hybrid**  **vaccination (Omicron convalescents) / Hybrid**  **vaccination (healthy donors)** | **WT** | 30.2 | 0.001 |
|  | **B.1.1.529** | 7.7 | 0.005 |
|  | **BA.5** | 6 | 0.02 |
|  | **BF.7** | 7.4 | 0.001 |
|  | **CH1.1** | 2.7 | 0.01 |

**Supplementary Table S4. Fold changes and p values between Delta cohort.**

|  |  | **Fold change** | **p value** |
| --- | --- | --- | --- |
| **Delta convalescents / Healthy donors** | **WT** | 8.6 | < 0.0001 |
|  | **Alpha** | 15.6 | < 0.0001 |
|  | **Beta** | 13.7 | < 0.0001 |
|  | **Delta** | 25.9 | < 0.0001 |
|  | **B.1.1.529** | 6.8 | 0.03 |
| **3^rd^ dose (healthy donors) / 2^nd^ dose (healthy donors)** | **WT** | 8.2 | 0.01 |
|  | **Alpha** | 4.1 | 0.59 |
|  | **Beta** | 1.7 | > 0.99 |
|  | **Delta** | 1.4 | > 0.99 |
|  | **B.1.1.529** | 1.4 | > 0.99 |
| **2^nd^ dose (Delta convalescents) / Unvaccinated (Delta convalescents)** | **WT** | 1.6 | 0.2 |
|  | **Alpha** | 2.2 | 0.75 |
|  | **Beta** | 2.1 | > 0.99 |
|  | **Delta** | 2.2 | 0.38 |
|  | **B.1.1.529** | 3.4 | 0.24 |
| **2^nd^ dose (Delta convalescents) / 2^nd^ dose (healthy donors)** | **WT** | 36.6 | < 0.0001 |
|  | **Alpha** | 68.2 | < 0.0001 |
|  | **Beta** | 29.7 | 0.0001 |
|  | **Delta** | 506 | < 0.0001 |
|  | **B.1.1.529** | 15.6 | < 0.0001 |

**Supplementary Table S5. Fold changes and p values** between Omicron inpatients with different vaccination backgrounds.

|  |  | **Fold change** | **p value** |
| --- | --- | --- | --- |
| **1^st^-2^nd^ dose / Unvaccinated** | **WT** | 2 | > 0.99 |
|  | **B.1.1.529** | 1.1 | > 0.99 |
|  | **BA.5** | 1 | > 0.99 |
|  | **BF.7** | 1.1 | > 0.99 |
|  | **CH1.1** | 1.8 | 0.23 |
| **3^rd^ dose / Unvaccinated** | **WT** | 9.5 | 0.2 |
|  | **B.1.1.529** | 2.8 | 0.98 |
|  | **BA.5** | 1 | > 0.99 |
|  | **BF.7** | 1.5 | > 0.99 |
|  | **CH1.1** | 3.3 | 0.02 |
| **4^th^ dose or hybrid**  **vaccination / Unvaccinated** | **WT** | 470.2 | 0.02 |
|  | **B.1.1.529** | 15 | 0.07 |
|  | **BA.5** | 10 | 0.15 |
|  | **BF.7** | 18.6 | 0.06 |
|  | **CH1.1** | 12.3 | 0.009 |
| **3^rd^ dose / 1^st^-2^nd^ dose** | **WT** | 4.8 | 0.6 |
|  | **B.1.1.529** | 2.6 | 0.82 |
|  | **BA.5** | 1 | > 0.99 |
|  | **BF.7** | 1.4 | > 0.99 |
|  | **CH1.1** | 1.9 | > 0.99 |
| **4^th^ dose or hybrid**  **vaccination / 1^st^-2^nd^ dose** | **WT** | 235 | 0.04 |
|  | **B.1.1.529** | 13.9 | 0.06 |
|  | **BA.5** | 10.3 | 0.11 |
|  | **BF.7** | 16.8 | 0.04 |
|  | **CH1.1** | 7 | 0.14 |
| **4^th^ dose or hybrid**  **vaccination / 3^rd^ dose** | **WT** | 49.4 | 0.94 |
|  | **B.1.1.529** | 5.3 | 0.92 |
|  | **BA.5** | 10.5 | 0.11 |
|  | **BF.7** | 12.4 | 0.23 |
|  | **CH1.1** | 3.7 | > 0.99 |

**Supplementary Table S6. Fold changes and p values** between different disease severity subgroups.

|  |  | **Fold change** | **p value** |
| --- | --- | --- | --- |
| **Survival Omicron inpatients / Non-severe convalescents** | **WT** | 33.6 | < 0.0001 |
|  | **B.1.1.529** | 3.8 | 0.0003 |
|  | **BA.5** | 6.2 | < 0.0001 |
|  | **BF.7** | 4.1 | 0.002 |
|  | **CH1.1** | 1.1 | > 0.99 |
| **Non-survival Omicron inpatients / Non-severe convalescents** | **WT** | 47.8 | < 0.0001 |
|  | **B.1.1.529** | 3.2 | 0.11 |
|  | **BA.5** | 4.6 | 0.0004 |
|  | **BF.7** | 2.7 | 0.02 |
|  | **CH1.1** | 1.8 | 0.15 |
| **Non-survival Omicron inpatients / Survival Omicron inpatients** | **WT** | 1.4 | > 0.99 |
|  | **B.1.1.529** | 0.8 | > 0.99 |
|  | **BA.5** | 0.7 | > 0.99 |
|  | **BF.7** | 0.7 | > 0.99 |
|  | **CH1.1** | 1.6 | 0.42 |

**Supplementary Table S7. AUC values with 95% CI in different subgroups.**

|  | **Healthy control**  **（n=9）** |  | | | **Omicron infected (n=33)** | | |
| --- | --- | --- | --- | --- | --- | --- | --- |
|  |  | **Omicron convalescents（n=16）** | | **Inpatient**  **(n=17)** | **Unvaccinated**  **(n=7)** | **Intramuscular**  **vaccine**  **(n=15)** | **Intramuscular and nasal spray vaccine**  **(n=11)** |
| **WT** | 19.9  (14.6-27.9) | 38.3  (19.3-58.4) | 32.8  (19.3-53.6) | | 51.0  (19.3-70.8) | 52.8  (19.5-58.4) | 20.4  (14.5-96.0) |
| **B.1.1.529** | 13.8  (12.1-22.8) | 27.8  (15.0-38.8) | 28.5  (19.0-40.7) | | 35.7  (19.0-74.0) | 28.5  (20.9-55.5) | 17.6  (12.4-61.7) |
| **BA.5** | 16.1  (9.3-23.7) | 20.9  (10.3-33.2) | 37.4  (30.4-53.2) | | 53.2  (27.6-70.3) | 30.8  (25.2-48.0) | 19.6  (9.0-65.5) |
| **BF.7** | 20.1  (9.9-24.7) | 29.0  (18.1-43.3) | 35.2  (28.9-45.6) | | 45.6  (18.5-125.5) | 31.3  (27.6-40.1) | 26.5  (15.1-113.3) |
